# Expansion of a circulating Ki67-positive effector T-cell population following combined PD-1 and CTLA-4 blockade for melanoma is predictive of treatment response

**DOI:** 10.1136/jitc-2025-012317

**Published:** 2025-10-09

**Authors:** Jack M Edwards, Sashendra Senthi, Robin Smith, Hayley Burridge, Carole Owens, Mark Shackleton, Miles C Andrews, Menno C van Zelm

**Affiliations:** 1 Department of Immunology, Monash University, Melbourne, Victoria, Australia; 2 Alfred Health Radiation Oncology, Alfred Hospital, Melbourne, Victoria, Australia; 3 Department of Medicine, Monash University, Melbourne, Victoria, Australia; 4 Department of Medical Oncology, Alfred Hospital, Melbourne, Victoria, Australia; 5 Department of Immunology, Erasmus MC, Rotterdam, The Netherlands

**Keywords:** Melanoma, Biomarker, T cell, Immune Checkpoint Inhibitor, Immunotherapy

## Abstract

**Background:**

Despite the success of combined cytotoxic T-lymphocyte associated protein 4 (CTLA-4) and programmed cell death protein-1 (PD-1) immune checkpoint blockade (cICB), the majority of patients with melanoma fail to respond or experience severe treatment-related toxicity. Currently, there are no reliable biomarkers available to predict these events and guide treatment choices. We here evaluated the peripheral immune compartment to identify features associated with cICB outcome and toxicity.

**Methods:**

Blood samples were collected from 51 patients with advanced melanoma prior to commencing and after one cycle of cICB. Patients were classified as responders or non-responders based on radiographic best overall response to treatment, and grouped by the occurrence of severe toxicity. Absolute immune cell counts were obtained and peripheral blood mononuclear cells were cryopreserved prior to spectral flow-cytometric T-cell immunophenotyping.

**Results:**

20 patients (39%) failed to respond to treatment, and 29 (57%) experienced severe toxicity. Pre-treatment, patients had fewer T cells than age-matched healthy controls (median 892 vs 1297 cells/µL, p=0.0004), mostly due to reduced naive CD4^+^ (p=0.0038) and CD8^+^ (p=0.0031) T cells. One cycle of cICB restored patient T cells to levels equivalent to healthy controls through expansion and activation of CD4^+^ and CD8^+^ memory and regulatory, but not naive subsets, and skewed the T-cell compartment towards an activated phenotype. This T-cell expansion correlated strongly with pre-treatment PD-1 (r=0.88, p=0.0003) but not CTLA-4 (r=0.32, p=0.34) expression levels, and was accompanied by upregulation of molecules including Ki67, inducible co-stimulator of T cells (ICOS), T-cell immunoglobulin and mucin-domain containing-3 (TIM-3), and T cell immunoreceptor with Ig and ITIM domains (TIGIT) on effector CD4^+^ and CD8^+^ T cells. Greater upregulation of Ki67 in CD4^+^ central memory cells significantly differentiated responders and non-responders after one cycle of treatment (p=0.0086, area under the curve (AUC)=0.74, 95% CI 0.59 to 0.88), while higher on-treatment TIM-3 frequency within CD8^+^ T cells differentiated patients who experienced severe toxicity (p=0.0086, AUC=0.74, 95% CI 0.59 to 0.88).

**Conclusions:**

We here show that response and toxicity to cICB in advanced melanoma are driven by distinct immune features evident after only one cycle of treatment. These could serve as prognostic biomarkers upon validation in larger cohorts.

**WHAT IS ALREADY KNOWN ON THIS TOPIC**

- Despite overall clinical success, combined programmed cell death protein-1 and cytotoxic T-lymphocyte associated protein immune checkpoint blockade is not effective in all patients and results in severe toxicity in up to 60%, and we do not fully understand the immune basis of these events nor have predictive biomarkers for them.

**WHAT THIS STUDY ADDS**

- This study finds that early on-treatment upregulation of the proliferative marker Ki67 in CD4^+^ central memory T cells associates with better response to treatment and longer progression-free survival, while the frequency of TIM-3 expression within CD8^+^ T cells associates with severe toxicity, raising the prospect that these outcomes are driven by distinct immunological features and may be uncoupled to improve treatment safety and efficacy.

**HOW THIS STUDY MIGHT AFFECT RESEARCH, PRACTICE OR POLICY**

- The findings presented here include potential prognostic biomarkers on validation in larger cohorts.

## Background

 Combined immune checkpoint blockade (cICB) targeting cytotoxic T-lymphocyte associated protein (CTLA-4) with ipilimumab and programmed cell death protein-1 (PD-1) with nivolumab is now standard of care for advanced melanoma and has shown success in neoadjuvant treatment of resectable disease.^1^ A 10-year follow-up of the CheckMate 067 trial showed that the median overall survival (OS) for patients with advanced melanoma receiving cICB with ipilimumab and nivolumab was 71.9 months, a significant improvement over treatment in the pre-ICB era (8 months),^2^ and monotherapy with either anti-PD-1 (36.9 months) or anti-CTLA-4 (19.9 months).^3^ Despite this success, 42% of patients experience no objective response to cICB, and up to 62% of patients experience severe treatment-related toxicity.^3^ There are currently no reliable predictors for these outcomes. Hence, there remains an unmet clinical need for reliable prognostic factors or predictive biomarkers of response and toxicity to cICB, which allow rational and personalized treatment decisions.

Given that cICB targets the immune system and toxicity mimics autoimmunity, systemic immune features prior to and during treatment likely influence outcome. Peripheral blood is readily and repeatedly accessible, contains tumor-specific immune cells, and can reflect changes that occur within the tumor,^4^,^6^ allowing ongoing monitoring of treatment response. Liquid biopsies thus represent a more promising source for biomarker discovery and evaluation of immune changes on treatment than tumor-based biomarkers, which are difficult to access and affected by intertumoral and intratumoral heterogeneity.^7^ Assessment of pre-treatment and early on-treatment time points allows for identification of features that are predictive for the clinical outcomes of interest.

While numerous studies have assessed immune features prior to and during treatment with PD-1 and/or CTLA-4 ICB, extracting reliable findings from these has been complicated by the aggregation of patients receiving different treatments or with tumors from variable tissue origin, variability in defining immune subsets, and heterogeneous findings between studies.^8^ In general, a higher absolute or relative abundance of lymphocytes, monocytes, or eosinophils, and a lower neutrophil abundance, have been associated with clinical benefit and/or improved OS following cICB across various cancers.^9^,^13^ It remains unclear which features associate with treatment-related toxicity, and whether these overlap with or are distinct from those associated with response; validation of putative toxicity biomarkers showed none were reliably predictive.^14^ Disentangling the features driving response and toxicity in real-world cohorts will enable safer application of cICB to those most likely to receive clinical benefit.

ICB functions by reinvigorating antitumor T-cell responses.^15^ Surface-expressed PD-1 and CTLA-4 on T cells function via distinct mechanisms to prevent overactivation of immune responses. CTLA-4 is upregulated following T-cell receptor engagement and outcompetes the costimulatory molecule CD28 in binding to CD80/86, acting early during T-cell activation in lymphoid tissues to dampen activation and proliferation. CTLA-4 blockade has been shown to increase CD8^+^ T-cell diversity,^16^ expand cytotoxic and ICOS^+^ Tbet^+^ Th1-like cells,^17^ and increase Ki67 expression in CD8^+^ T cells.^18 19^ The PD-1 binding partners programmed death-ligand 1 (PD-L1) and PD-L2 are widely expressed on non-lymphoid tissues in response to inflammatory cytokines, and so PD-1 primarily acts at the effector stage of established immune responses.^20^ PD-1 blockade expands existing effector and exhausted CD8^+^ T cells,^4 18 21^ and increases proliferation of regulatory T cells (Treg).^22^ Notably, preferential activation of effector T cells rather than Treg associates with treatment benefit.^23^

Thus, the primary target of ICB is exhausted CD8^+^ T cells (Tex), which display diminished functional and proliferative capacity in response to repeated antigen exposure or manipulation by the tumor.^24^ Tex are enriched in tumors and typically defined by co-expression of multiple inhibitory molecules including PD-1, TIGIT, TIM-3, and lymphocyte-activation gene 3 (LAG-3).^18^ Conversely, precursor (or progenitor) Tex cells (Tpex) expressing TCF1 retain proliferative potential, and expand in response to PD-1 blockade to repopulate CX3CR1^+^ transitory/effector and terminally exhausted PD-1^+^ TIM-3^+^ Tex cells,^25 26^ and are important for therapeutic efficacy.^27 28^ An increased proportion of the TCF1^+^ subset within PD-1^+^ CD8^+^ tumor-infiltrating T cells has been associated with longer progression-free survival (PFS),^29^ and TCF1^+^ T cells are increased in ICB-treated patients,^30^ but are not consistently different between responders (R) and non-responders (NR).^29 31^

The effects of cICB on PD-1^+^ T cells remain to be fully elucidated, with conflicting reports of cICB both increasing or decreasing the frequencies of TCF1^+^ Tpex early on-treatment.^18 19^ Compared with PD-1 or CTLA-4 monotherapy, cICB appears to synergistically drive greater Ki67 expression in Tpex and Tex,^18^ potentially explaining the increased therapeutic efficacy of cICB. However, this work was performed in a small cICB cohort of nine patients skewed towards responders, and did not assess the role of CD4^+^ T cells or their association with treatment response.^18^ However, the upregulation of Ki67, which peaks 1–6 weeks post-commencement of cICB,^1821 22 32^,^34^ also occurs in PD-1^−^ T cells,^4 19^ and has been associated with treatment outcome in various cancers.^4 35 36^ In tandem, cICB expands central and effector memory T cells, and these have been associated with positive treatment outcome.^2134 37^,^39^ These expanded populations of Ki67^+^ and memory T cells may hence constitute the effector cells of cICB, but it remains unclear whether treatment expands an existing pool of functional cells or also enhances their functionality. Further work to determine the phenotype of these cells and their association with response and toxicity to cICB in advanced melanoma is warranted.

To address this, we evaluated pre-treatment and on-treatment systemic immunity in a cohort of patients with advanced melanoma commencing cICB and found that response and toxicity were associated with distinct immune features. Responders had significantly higher Ki67 upregulation in CD4^+^ central memory T cells compared with non-responders, while patients experiencing toxicity had significantly higher on-treatment numbers of TIM-3^+^ CD8^+^ T cells.

## Methods

### Patients and healthy volunteers

Patients commencing combined ipilimumab and nivolumab systemic therapy for the treatment of unresectable stage III or IV melanoma at the Alfred Hospital, Melbourne, Australia, were eligible for inclusion and recruited to The James Foster Foundation Alfred Cancer Biobank under protocols approved by the Alfred Ethics Committee. Written informed consent was obtained from all participants prior to enrollment. All patients received the standard advanced disease melanoma-dosing regimen: ipilimumab 3 mg/kg+nivolumab 1 mg/kg intravenously 3 weekly for up to four cycles, followed by maintenance nivolumab 3 mg/kg or 480 mg (flat-dosing) 4 weekly. Blood samples were obtained pre-treatment and on-treatment (immediately prior to cycle 2), and stored peripheral blood mononuclear cell (PBMC) were retrieved from the biobank under the translational research in immunotherapy patients study (AEC 507/20). Age-matched healthy controls (HC) were recruited under an approved protocol from Monash University (26385). All studies were conducted in accordance with the ethical principles of the Declaration of Helsinki and with adherence to the Good Clinical Practice guidelines as defined by the International Conference on Harmonization.

### Sample processing

Blood samples were collected in Vacutainer K2EDTA blood tubes (BD Biosciences, San Jose, California, USA). Total leukocyte counts were determined with the Cell Dyn analyzer (Abbott Core Laboratory, Abbott Park, Illinois, USA), and in addition, 50 µL was taken for flow cytometric TruCount analysis (see below). Blood samples were centrifuged at 1,320 g for 10 min, plasma was removed and centrifuged again at 12,000 g for 10 min before storage at −80°C. The remaining blood was diluted 1:1 with phosphate-buffered saline (PBS) and loaded onto Ficoll-Hypaque Plus (Cytiva, Marlborough, Massachusetts, USA) for isolation of PBMCs by density centrifugation at 650 g for 25 min with no brake. After washing two times with PBS, PBMCs were cryopreserved in liquid nitrogen at a density of 10 million viable cells/mL in 50% fetal calf serum (FCS; Bovogen, Melbourne, VIC, Australia), 40% RPMI-1640 (Sigma-Aldrich, St. Louis, Missouri, USA), and 10% dimethyl sulfoxide (DMSO; Sigma-Aldrich).

### TruCount analysis

Absolute numbers of leukocytes, granulocytes, monocytes, lymphocytes, and subsets therein were determined using a lyse-no-wash T, B and NK TruCount assay, as previously described.^40^ Briefly, within 6 hours of sample collection, 50 µL of whole blood was added to a TruCount tube (BD Biosciences) together with an antibody cocktail of 20 µL containing conjugated primary antibodies against human CD3, CD4, CD8, CD14, CD16, CD19, CD45, CD56 and HLA-DR (online supplemental table 1). Following incubation for 15 min at room temperature, samples were lysed with FACS Lysing Solution (BD Biosciences) in a total volume of 500 µL for 15 min and stored at 4°C in the dark for up to 1 hour before acquisition on a Fortessa LSRII or FACSLyric flow cytometer (BD Biosciences).

### T-cell immunophenotyping

Cryopreserved PBMCs were batch-thawed and stained using two previously described spectral flow cytometry panels for in-depth phenotyping of T cells.^40^ One tube was designed to interrogate surface and intracellular marker expression by resting ex vivo T cells, and a second to evaluate the production of effector molecules after overnight stimulation with CD3 and CD28 in the presence of brefeldin A and monensin. The panel composition and antibody details are listed in online supplemental table 3. Samples were acquired on a 5-laser Cytek Aurora with standard Cytek Assay Settings adjusted daily using SpectroFlo quality control (QC) beads (Cytek Biosciences, Fremont, California, USA) as per the manufacturer’s recommendations. Forward scatter (FSC) and side scatter (SSC) were adjusted to optimally identify the lymphocyte population, and the FSC area scaling factor was set to 0.95. Samples were acquired using the live unmixing functionality and run at a medium flow rate, averaging 3,000–5,000 events/s. A median of 605,760 viable T cells was assessed per patient per time point across the two spectral flow cytometry panels.

### Data analysis

**Figure 1:**
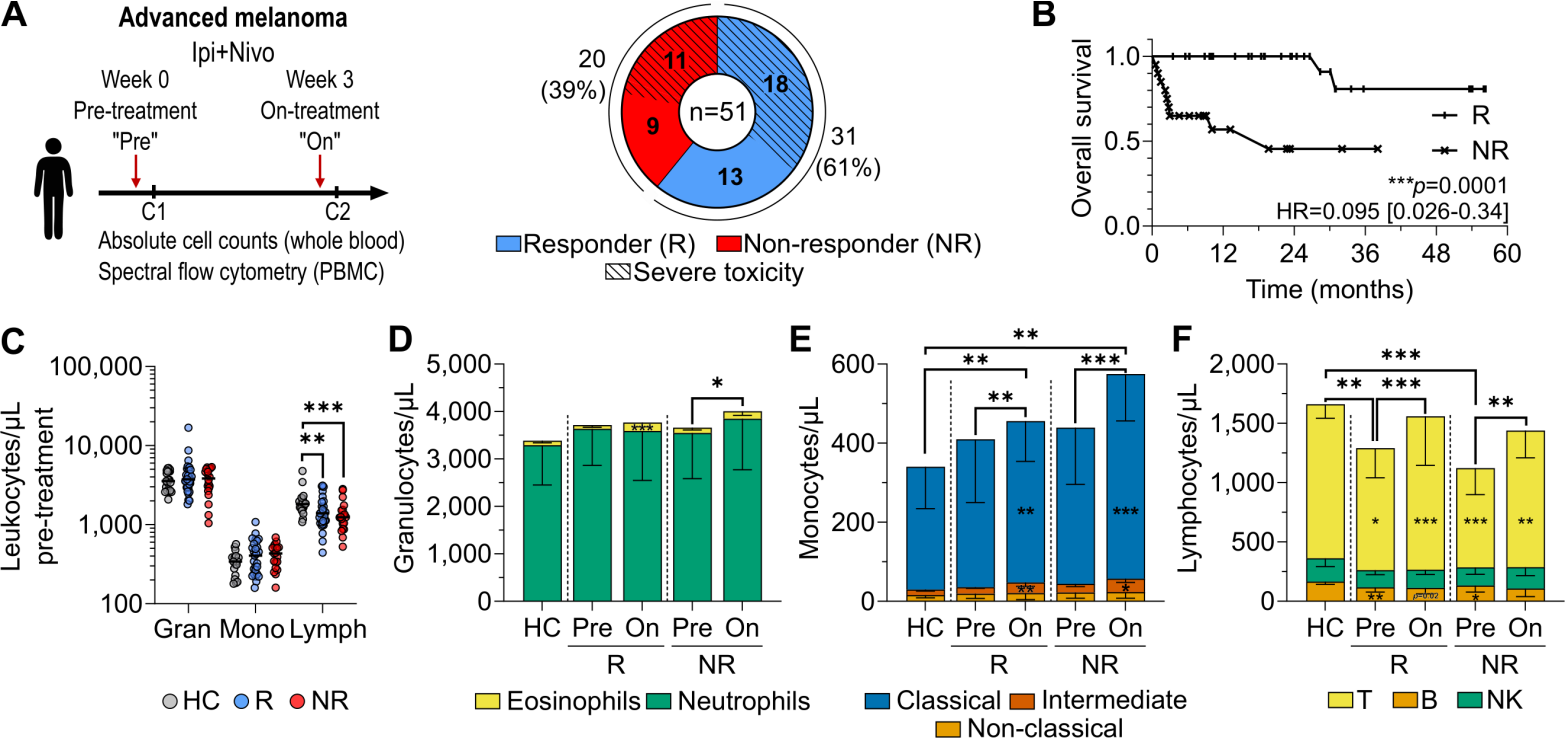
Clinical features of patient cohort and effects of cICB on innate immune cells. (**A**) Blood sampling scheme, and response and toxicity outcomes of the patient cohort. (**B**) Kaplan-Meier overall survival of responders (R) versus non-responders (NR). (**C**) Pre-treatment absolute counts of granulocytes (gran), monocytes (mono), and lymphocytes (lymph) in healthy controls (HC), R, and NR prior to cICB. Pre-treatment and on-treatment absolute counts of (**D**) neutrophils and eosinophils, (**E**) classical, intermediate and non-classical monocytes, and (**F**) T, B, and natural killer cells. Asterisks within the pre-treatment bars denote significant differences compared with HC, while asterisks within on-treatment bars denote significant pre-treatment to on-treatment changes, and asterisks above denote significant differences in total (**D**) granulocyte, (**E**) monocyte, or (**F**) lymphocyte counts. Plots show median±IQR range (IQR). Data shown from 21 controls, 31 pre-treatment and on-treatment responders, and 20 pre-treatment and 16 on-treatment non-responders. Statistical comparison of HC, R, and NR used the unpaired Kruskal-Wallis test, with subsequent direct comparisons using the Mann-Whitney test. Paired pre-treatment to on-treatment comparisons were evaluated with the Wilcoxon signed-rank test; Bonferroni correction for multiple comparisons was applied to all tests. *p<0.0167, **p<0.01, ***p<0.001, ****p<0.0001. cICB, combined immune checkpoint blockade; PBMC, peripheral blood mononuclear cell.

**Figure 2:**
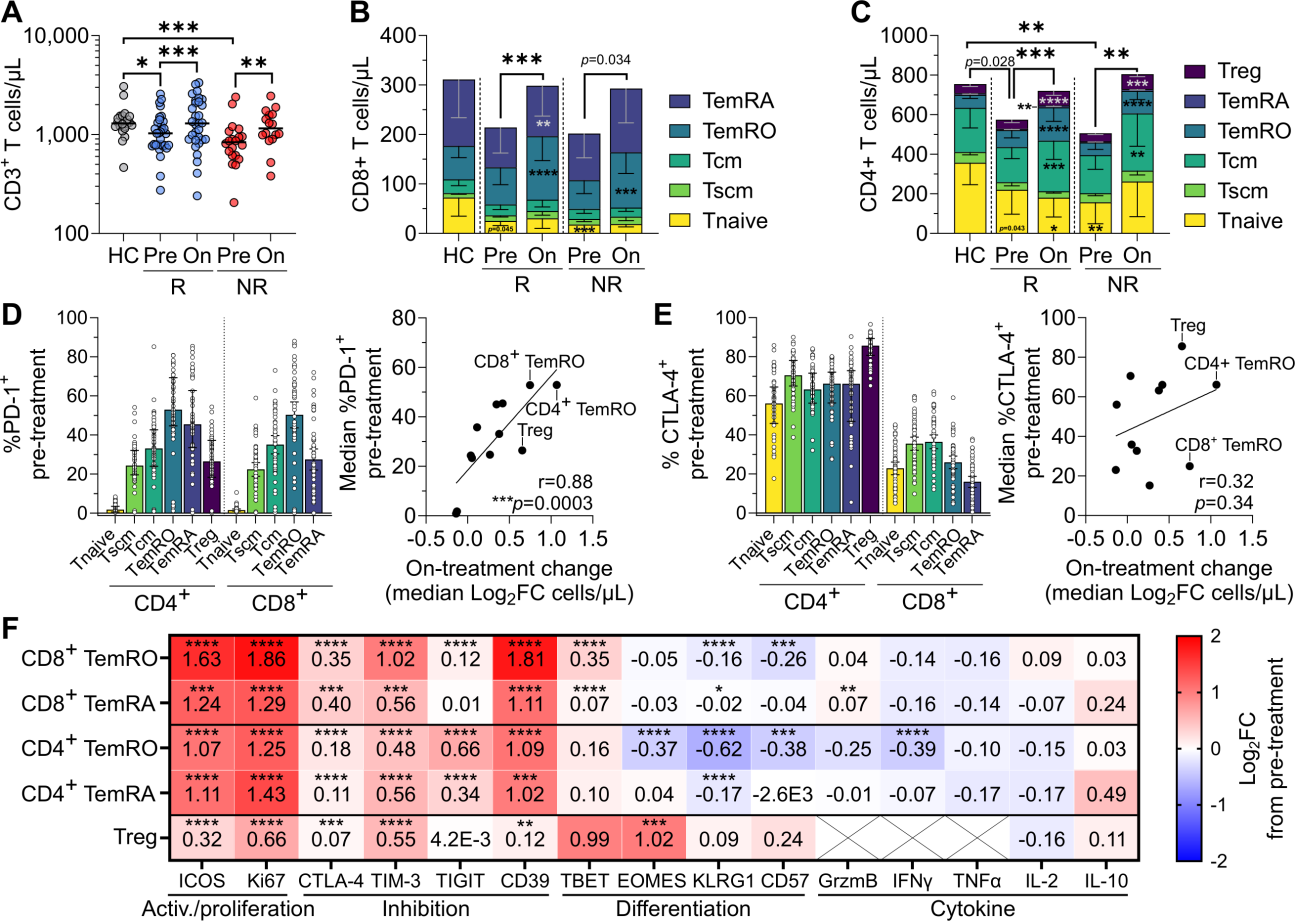
On-treatment T-cell recovery is driven by expanded CD4^+^ and CD8^+^ effector and regulatory subsets. (**A**) Total T-cell numbers in healthy controls (HC), and before and after one cycle of combined immune checkpoint blockade in responders (R) and non-responders (NR). (**B**) CD8^+^ and (**C**) CD4^+^ T-cell subset abundance in HC, R, and NR. Asterisks within the pre-treatment bars denote significant differences compared to HC, while asterisks in the on-treatment bars denote significant pre-treatment to on-treatment changes, while asterisks above denote significant differences in total (**B**) CD8^+^ or (**C**) CD4^+^ T-cell counts. (**D**) Per cent of T-cell subsets expressing PD-1, and the correlation between subset median PD-1 expression and on-treatment fold change in abundance in all patients. (**E**) Per cent of T-cell subsets expressing CTLA-4, and the correlation between subset median CTLA-4 expression and on-treatment fold change in abundance in all patients. (**F**) Pre-treatment to on-treatment median log_2_ fold change of activation, inhibition, differentiation, and cytokine marker positivity for CD8^+^ and CD4^+^ TemRO and TemRA subsets, and CD4^+^ Treg in all patients. Crosses indicate the marker was not assessed. Plots show medians±IQR. Data shown from 21 controls, 31 pre-treatment and on-treatment responders, and 20 pre-treatment and 16 on-treatment non-responders. Unpaired and paired statistical comparisons were performed with the Mann-Whitney and Wilcoxon signed-rank tests, respectively; Bonferroni correction for multiple comparisons was applied to all tests *p<0.0167, **p<0.01, ***p<0.001, ****p<0.0001. CD39, cluster of differentiation 39; CTLA-4, cytotoxic T-lymphocyte associated protein 4; EOMES, eomesodermin; GrzmB, granzyme B; ICOS, inducible co-stimulator of T cells; IFN, interferon; IL, interleukin; KLRG1, killer cell lectin-like receptor subfamily G member 1; log_2_FC, log_2_ fold change; PD-1, programmed cell death protein-1; TBET, T-box expressed in T cells; Tcm, central memory; TemRO/RA, T effector memory expressing CD45RO/RA; TIGIT, T cell immunoreceptor with Ig and ITIM domains; TIM-3, T-cell immunoglobulin and mucin-domain containing-3; Tnaive, naive T cell; TNF, tumor necrosis factor; Tscm, stem cell-like memory; Treg, regulatory T cells.

**Figure 3:**
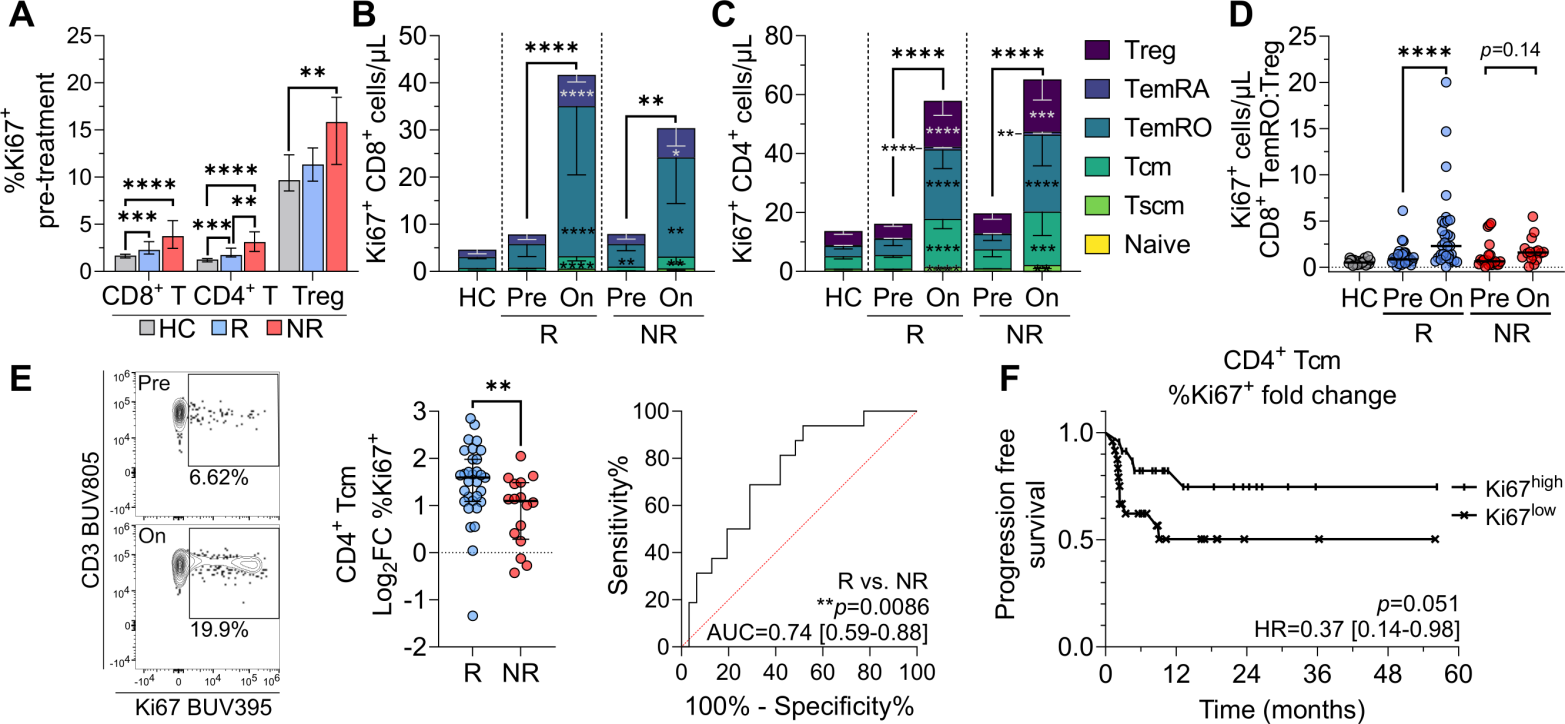
Greater on-treatment expansion of Ki67^+^ T cells differentiates responders and non-responders. (**A**) Pre-treatment per cent Ki67 positivity in CD8^+^ T, CD4^+^ T, and Treg cells. Absolute number of Ki67^+^ (**B**) CD8^+^ and (**C**) CD4^+^ T subsets in healthy controls (HC), responders (R), and non-responders (NR). Asterisks within the pre-treatment bars denote significant differences compared to HC, while asterisks in on-treatment bars denote significant pre-treatment to on-treatment changes, and asterisks above denote significant differences in total Ki67^+^ CD8^+^ or CD4^+^ T cells. (**D**) Pre-treatment and on-treatment ratio of Ki67^+^ CD8^+^ TemRO to Ki67^+^ Treg cell count. (**E**) Pre-treatment to on-treatment Log_2_ fold change in Ki67 positivity within CD4^+^ Tcm cells, and responder versus non-responder receiver operator characteristic (ROC) curve with area under the curve (AUC) and 95% CI noted. (**F**) Kaplan-Meier progression-free survival in patients with high (>median) or low (<median) pre-treatment to on-treatment Log_2_FC of Ki67 positivity in CD4^+^ Tcm cells. Plots show medians±IQR. Data shown from 21 controls, 31 pre-treatment and on-treatment responders, and 20 pre-treatment and 16 on-treatment non-responders. Statistical comparison of HC, R, and NR used the unpaired Kruskal-Wallis test, with subsequent direct comparisons using the Mann-Whitney test. Paired pre-treatment to on-treatment comparisons were performed with the Wilcoxon signed-rank test; Bonferroni correction for multiple comparisons was applied to all tests. *p<0.0167, **p<0.01, ***p<0.001, ****p<0.0001. Log_2_FC, log_2_ fold change; Tcm, central memory; TemRO/RA, T effector memory RO/RA; Tnaive, naive T cell; Tscm, stem cell-like memory; Treg, regulatory T cell.

All samples were analyzed using FlowJo V.10.10 (BD Biosciences), with minor compensation adjustments performed as required after fluorochrome-specific biexponential scaling. Only samples with high cell viability (>80%) and stability (assessed by tracking the CD3 BUV805 intensity vs time) were included in this study. Manual identification of immune cell populations was performed as previously described for each of the TruCount, resting, and activated panels (online supplemental tables 1,4,5 and figures 1–3).^40^ Using the absolute cell numbers obtained from the TruCount panel, T-cell subset frequencies were converted into absolute counts per µL of blood; to avoid interdependence of subset proportions, we typically chose to report the absolute count of subsets and frequencies of marker expression therein. Data were analyzed and plotted using GraphPad Prism V.10.4.1, and the tidyverse V.2.0.0 and ggpubr V.0.6.0 packages in the R programming environment V.4.2.2^41^,^43^ were used for Kruskal-Wallis, Mann-Whitney, and Wilcoxon signed-rank statistical tests as below.

### Statistical analysis

Non-parametric statistical analyses were performed using the Mann-Whitney test for comparison between two independent groups, the Kruskal-Wallis test for multiple independent groups, and the Wilcoxon signed-rank test for two paired groups. Considering the hierarchical, dependent nature of the data, multiple comparisons adjustment was performed using the Bonferroni method with respect to three major T-cell lineages: γδT, CD8^+^ T, and CD4^+^ T cells, leaving an adjusted significance threshold (alpha) for all tests of 0.0167. Differences observed at the lineage level were then explored in the dependent subsets within. GraphPad Prism V.10.1.2 was used to perform log-rank (Mantel-Cox) survival, receiver operator characteristic, Spearman non-parametric correlation, multiple logistic regression, Cox proportional hazards regression, and Fisher’s exact tests. P values <0.0167 were considered significant. Patients with missing on-treatment samples were not included in paired statistical analyses, and were removed from survival analyses using on-treatment data.

## Results

### Patient cohort and clinical features

51 patients with unresectable stage III or IV melanoma scheduled to be treated with combined ipilimumab and nivolumab ICB were recruited between November 2020 and February 2025, and peripheral blood samples were collected prior to and on treatment (table 1, figure 1A). On-treatment samples were unavailable for four patients, who were all non-responders, allowing analysis of 51 pre-treatment and 47 on-treatment samples. The median age at inclusion was 63 years, 68% of patients were men, and 53% were treatment naive. Patients were grouped for analysis by best overall response to treatment under Response Evaluation Criteria in Solid Tumors (RECIST) V.1.1 guidelines.^44^ After a median follow-up time of 790 days, 31 patients (61%) experienced a partial or complete response to treatment (responders; R), and the remaining 20 patients had stable or progressive disease, or passed away (non-responders; NR). Treatment-related toxicity was identified through clinicopathologic assessment by treating clinicians and graded according to the Common Terminology Criteria for Adverse Events V.5.0.^45^ Responders were more likely to experience any grade of treatment-related toxicity (p=0.0066); however, severe toxicity (≥grade 3) occurred at similar rates in responders (58%) and non-responders (55%) (figure 1A). Non-responders were slightly more likely to have received prior radiotherapy (p=0.042). Sex, age, *BRAF*/*NRAS* mutation status, metastatic disease site(s), pre-treatment performance score (Eastern Cooperative Oncology Group (ECOG)),^46^ lactate dehydrogenase, or prior therapies were not otherwise different between responders and non-responders (table 1). Median OS was significantly higher for responders (not reached) than for non-responders (19.7 months) (p=0.0001) (figure 1B), but did not differ based on the occurrence of severe toxicity (online supplemental figure 4A, table 1). The observed association between OS and the occurrence of any-grade toxicity (p<0.0001) is likely an artifact due to the low number of patients not experiencing toxicity (n=4). Besides an association between stage IIIC/IID and poor OS (p=0.036) (online supplemental figure 4B), there were no associations between clinical features and OS, likely due to low patient numbers (table 1). 21 healthy controls were also included with a matched median age of 62 years (range 39–81), of which 57% were women.

**Table 1:** Pre-treatment clinical and immunological characteristics of patients with advanced melanoma

	All patients (n=51)	Responders (n=31)	Non-responders (n=20)	P value (R vs NR)*	P value (OS)†
Age (years)					
Median (range)	62 (25–81)	62 (25–79)	65 (26–81)	0.35‡	0.88
Sex					
Male	35 (69%)	20 (65%)	15 (75%)	0.54	0.19
Female	16 (31%)	11 (35%)	5 (25%)		
Toxicity (CTCAE V.5.0)§					
Any grade	46 (90%)	31 (100%)	15 (75%)	0.0066	<0.0001
Grade 3+	29 (57%)	18 (58%)	11 (55%)	>0.99	0.75
Median time (days) to onset of severe toxicity (range)	46 (12–198)	51 (27–119)	42 (12–198)	0.07	0.33
Genomics					
BRAF V600 mutant	19 (37%)	9 (29%)	10 (50%)	0.35	0.15
NRAS mutant	15 (30%)	10 (32%)	5 (25%)		
BRAF/NRAS wt	17 (33%)	12 (39%)	5 (25%)		
Stage of disease (AJCC eighth ed.)§					
Stage III (C–D)	4 (8%)	1 (3%)	3 (15%)	0.29	0.036
Stage IV (total)	47 (92%)	30 (97%)	17 (85%)		
IV A	6 (12%)	5 (16%)	1 (5%)		
IV B	3 (6%)	3 (10%)	0 (0%)		
IV C	16 (31%)	9 (29%)	7 (35%)		
IV D	22 (43%)	13 (42%)	9 (45%)		
Metastatic disease sites					
Brain	22 (43%)	13 (42%)	9 (45%)	>0.99	0.53
Liver	15 (30%)	8 (26%)	7 (35%)	0.58	0.71
Multi-site involvement (≥3 sites)	27 (53%)	18 (58%)	9 (45%)	0.40	0.82
Pre-treatment LDH§					
Elevated (>upper limit of normal)	25 (49%)	18 (58%)	7 (35%)	0.13	0.59
Normal	15 (30%)	9 (29%)	6 (30%)		
Not measured	11 (21%)	4 (13%)	7 (35%)		
ECOG performance status score at baseline§					
0	30 (59%)	19 (61%)	11 (55%)	0.62	0.14
1	20 (39%)	12 (39%)	8 (40%)		
Unknown	1 (2%)	0 (0%)	1 (5%)		
Pre-treatment ratios; median (range)					
Neutrophil:lymphocyte	2.57 (1.11–8.70)	2.62 (1.13–7.31)	2.35 (1.11–8.70)	0.49‡	0.79
Lymphocyte:monocyte	3.64 (1.34–10.58)	3.86 (1.34–6.31)	3.20 (1.57–10.58)	0.26‡	0.61
Prior therapy					
Treatment naive	27 (53%)	18 (58%)	9 (45%)	0.40	0.47
Immunotherapy	15 (30%)	9 (29%)	6 (30%)	>0.99	0.53
Targeted therapy	7 (14%)	3 (10%)	4 (20%)	0.41	0.48
Radiotherapy	12 (24%)	4 (13%)	8 (40%)	0.042	0.18
Cytotoxic	1 (2%)	0 (0%)	1 (5%)	0.39	–

* Fisher’s exact test, except as noted.

† Log-rank (Mantel-Cox) test (discrete data) or Cox proportional hazards regression (continuous data).

‡ Mann-Whitney test.

§ CTCAE; Common Terminology Criteria for Adverse Events V.5.0,^45^ AJCC; American Joint Committee on Cancer,^65^ ECOG; Eastern Cooperative Oncology Group.^46^

LDH, lactate dehydrogenase; NR, non-responder; OS, overall survival; R, responder; wt, wild type.

### One cycle of cICB drives recovery of lymphocyte numbers back to healthy control levels

Pre-treatment, total leukocyte counts were not significantly different between responders (median 5,653 cells/µL), non-responders (6,041 cells/µL), and healthy controls (5,900 cells/µL) (p=0.76) (online supplemental figure 4C). However, lymphocyte numbers were lower in both responders (1,389 cells/µL, p=0.0035) and non-responders (1,242 cells/µL, p=0.00098) than in age-matched healthy controls (1,824 cells/µL) (figure 1C), but this did not associate with OS when assessed by Cox proportional hazards regression (HR=1.00, 95% CI 0.99 to 1.00). Both granulocyte and monocyte lineages and subsets within were not different pre-treatment, but monocytes expanded on-treatment in all patients (figure 1D,E). Eosinophils expanded only in responders (p=0.00011), whereas expanding neutrophils drove increased total granulocyte counts in non-responders (p=0.011) (figure 1D). Responders had a significantly higher pre-treatment neutrophil:lymphocyte ratio (NLR) than healthy controls (p=0.0011), and both responders (p=0.0002) and non-responders (p=0.0012) had a lower lymphocyte:monocyte ratio (LMR) than healthy controls (online supplemental figure 4D,E). However, there were no significant differences between responders and non-responders, and neither pre-treatment NLR, LMR, nor neutrophil:eosinophil ratio (NER) was associated with OS in this cohort (online supplemental figure 4D–F, and table 1). Given that patients, and especially non-responders, had significantly lower lymphocyte counts pre-treatment, we sought to explore this lineage further and determine whether cICB could recover this phenotype back to levels equivalent to healthy controls.

### Expansions of regulatory T cells and CD4^+^ and CD8^+^ T memory cells on treatment

We noted that the decreased pre-treatment patient lymphocyte counts were predominantly due to lower total T-cell numbers in responders (1,029 cells/µL, p=0.010) and non-responders (837 cells/µL, p=0.00014) than in healthy controls (1,297 cells/µL), but that one cycle of cICB was able to rapidly recover conventional T-cell numbers in patients back to levels equivalent to controls (figure 2A). This expansion represented a log_2_ fold change (log_2_FC) increase in T-cell counts of 0.31 for responders (p=0.00012) and 0.41 (p=0.0052) for non-responders. B, natural killer (NK) and γδT cells generally did not increase on-treatment (online supplemental figure 5A–C). To examine which subsets were deficient pre-treatment, and if these recovered on-treatment, we performed an in-depth assessment of T-cell phenotype using two recently optimized spectral flow cytometry panels (40). With these panels, we assessed the expression of 12 phenotyping markers and 7 cytokines across 17 distinct T-cell subsets (online supplemental tables 3–5). We enumerated γδT and αβT cells; CD4^+^, CD8^+^, and CD4^−^CD8^−^ T cells; CD4^+^ Treg (CD127^low^CD25^+^) and follicular helper T cells (CD45RA^−^CXCR5^+^ Tfh); and naive (CD45RA^+^CCR7^+^CD95^−^ Tnaive), stem cell-like memory (CD45RA^+^CCR7^+^CD95^+^ Tscm), central memory (CD45RA^−^CCR7^+^ Tcm), effector memory (CD45RA^−^CCR7^−^ TemRO), and effector memory re-expressing CD45RA (CD45RA^+^CCR7^−^ TemRA) within CD4^+^ and CD8^+^ T subsets.^40 47^

Pre-treatment, patients had a median of 31% fewer circulating T cells than healthy controls (figure 2A). This was predominantly due to fewer CD8^+^ Tnaive (p=0.0050) and CD4^+^ Tnaive cells (p=0.0069), the latter of which contributed to a significantly lower total CD4^+^ T-cell count (p=0.0053) (figure 2B,C). This led to a proportional increase in Treg in both responders (p=0.0069) and non-responders (p=0.00046; online supplemental figure 5E). As absolute numbers of Treg were not increased (figure 2C), this proportional expansion was not intrinsic to Treg, but an indirect effect of the reduced numbers of Tnaive, demonstrating the importance of assessing both absolute and proportional data. While T-cell subset numbers did not differ significantly between response groups pre-treatment, non-responders tended to have even further reduced total and naive T-cell numbers than responders (figure 2A–C).

The increase in T-cell numbers after one cICB cycle resulted mostly from an expansion of memory and regulatory cells, particularly CD4^+^ and CD8^+^ Tcm, TemRO, and TemRA, whereas Tnaive numbers remained unchanged (figure 2B,C; online supplemental table 6). T memory subset expansion seemed agnostic of treatment outcome.

Notably, the magnitude of T-cell expansion correlated significantly with pre-treatment expression of PD-1, but not CTLA-4, implying that PD-1 blockade is primarily responsible for driving or potentiating the increased abundance of memory and regulatory subsets (figure 2D,E). Pre-treatment expression of PD-1 and CTLA-4 did not differ between responders, non-responders, and healthy controls, nor did the Boolean co-expression of the inhibitory receptors PD-1, TIM-3, and TIGIT on CD8^+^ T cells (online supplemental figure A–C), reflecting the similar expansion dynamics between responders and non-responders. However, CD39, an ectonucleotidase crucial for the production of the immunosuppressive molecule adenosine and a proposed marker of terminal exhaustion in CD8^+^ T cells,^48^ was expressed by a higher proportion of total CD8^+^ T, TemRO, and TemRA cells pre-treatment in patients versus healthy controls (online supplemental figure 6B,D). Both responders and non-responders experienced similar increases in memory T-cell and Treg subset abundance after one cycle of cICB.

### Increased expression of activation and inhibitory markers on expanded T-cell populations

As cICB drives expansion of memory and Treg and these are reported to harbor tumor-specific clones,^4 19^ we assessed if treatment induced differential changes in effector and regulatory subset phenotypes. As observed previously for cICB,^17^ we noted a pharmacodynamic increase in ICOS and TBET expression across CD8^+^ and CD4^+^ memory and Treg cells (figure 2F). Concomitantly, Th1 cells increased nearly twofold in responders (from 25 to 45 cells/µL, p=0.00081), but not in non-responders. Tfh, Th2, and Th17 counts generally increased following cICB in all patients (online supplemental figure 5F). Alongside ICOS, the proliferative marker Ki67 showed the highest on-treatment increase in expression (figure 2F), the magnitude of which was significantly greater in effector T cells than in Treg.

The inhibitory receptors TIM-3, CTLA-4, and CD39 were also upregulated across CD4^+^ and CD8^+^ T cells (figure 2F). The inhibitory receptor TIGIT was significantly upregulated only in CD4^+^ T cells, likely because 70%–80% of CD8^+^ TemRO/RA already expressed TIGIT pre-treatment. The exhaustion-related transcription factor TOX tended to be expressed at lower levels in CD8^+^ TemRO of responders pre-treatment, but increased on-treatment (p=0.000045) to match non-responders. In line with this, CD8^+^ T-cell expression of CD127 decreased on-treatment (online supplemental figure 6E,F). KLRG1 and the senescence marker CD57 were downregulated on CD8^+^ and CD4^+^ TemRO following cICB. There were few significant changes in the production of effector molecules including granzyme B, interferon (IFN)-γ, and tumor necrosis factor (TNF)-α, after in vitro stimulation of T cells with CD3 and CD28, besides a decrease in IFN-γ production by CD4^+^ TemRO (figure 2F). Hence, cICB expanded a subset of activated effector memory T cells that had increased expression of both activation and inhibitory markers, but no apparent change to the expression of cytolytic markers.

Interleukin (IL)-2 produced by CD8^+^ cells has been shown to expand Treg within the tumor via induction of ICOS,^49^ and we here observed an increased production of IL-2 by CD8^+^ TemRO, and expansion of Treg cells with increased ICOS expression (figure 2C,F). However, these features did not directly correlate. Notably, the proportion of Treg expressing the immunosuppressive enzyme CD39 increased only in responders (p=0.0031) and on-treatment. Treg had increased activation and inhibitory marker expression akin to effector subsets, albeit at a lower magnitude (figure 2F). This included ICOS, a marker of highly suppressive Tregs capable of suppressing intratumoral CD8^+^ T-cell maturation.^50^ Hence, cICB induced dynamic changes to circulating Treg but appeared to have a more pronounced impact on effector memory cells. We posit that an enrichment of proliferative effector over regulatory T cells will associate with better treatment outcome.

### Increased Ki67 in CD4^+^ Tcm on cICB differentiates responders and non-responders and associates with overall survival

Given that Ki67 was the most upregulated marker in effector memory T cells and highly upregulated in Treg following one cycle of cICB, we focused more closely on Ki67^+^ cells to determine whether they were associated with treatment outcome. We found that pre-treatment, significantly higher proportions of T cells expressed Ki67 in both responders (median 2.28% and 1.73% Ki67^+^ for total CD8^+^ and CD4^+^ T cells, respectively) and non-responders (3.74% and 3.12%) than in healthy controls (1.67% and 1.26%). In comparison, only non-responders had significantly higher frequencies of Treg expressing Ki67 (15.8%) than healthy controls (9.7%) (figure 3A). Mirroring this, pre-treatment activated ICOS^+^ Treg frequencies were elevated in non-responders over both responders (p=0.011) and healthy controls (p=0.04) (online supplemental figure 7A).

In addition, cICB had differential effects on responders versus non-responders. The absolute numbers of circulating Ki67^+^ cells increased significantly on treatment for both CD8^+^ (figure 3B) and CD4^+^ (figure 3C) T-cell lineages. Ki67^+^ Tcm and TemRO cells had greater on-treatment expansion than Treg; furthermore, this expansion was consistently higher in responders than non-responders. While the overall ratio of CD8^+^ TemRO to Treg did not change on treatment (online supplemental figure 7B), within the Ki67^+^ pool cICB enriched for CD8^+^ TemRO cells over Treg, indicating that treatment preferentially targeted proliferative effector over regulatory subsets. Notably, this was only significant in responders (figure 3D). Indeed, on-treatment fold increases in the frequency of Ki67^+^ cells trended higher in responders across all CD4^+^ and CD8^+^ subsets (online supplemental figure 7C,D). Of these, the on-treatment increase in Ki67^+^ CD4^+^ Tcm frequencies was significantly higher in responders than non-responders (p=0.0079); responder CD4^+^ Tcm cells increased from 2.5% Ki67^+^ pre-treatment to 7.1% on-treatment, while non-responders increased from 3.2% to 6.5%. This fold increase significantly differentiated response groups after only one cycle of treatment (area under the curve (AUC)=0.74, p=0.0086) (figure 3E). CD8^+^ Ki67^+^ Tcm and TemRO features were less predictive (online supplemental figure 7E,F). Among the clinical features evaluated in the univariate analysis, prior radiotherapy was the most significant difference between response groups (p=0.042, table 1). A multiple logistic regression model incorporating the fold change in Ki67^+^ CD4^+^ Tcm cell frequency and prior radiotherapy slightly improved this predictive capacity (AUC=0.75, 95% CI 0.61 to 0.90, p=0.0050) (online supplemental figure 7G), and a higher fold change in Ki67^+^ CD4^+^ Tcm cells tended to still associate with response (OR=0.45, 95% CI 0.17 to 1.01, p=0.069). Regardless of response outcome, patients with a higher Log_2_FC in Ki67 CD4^+^ Tcm cells (>median value of 1.32) tended to experience longer PFS (p=0.051) (figure 3F). Overall, this suggests that the magnitude of memory T-cell activation, particularly CD4^+^ Tcm, is predictive of clinical benefit from cICB.

### cICB expands an already functional pool of Ki67^+^ effector cells

**Figure 4:**
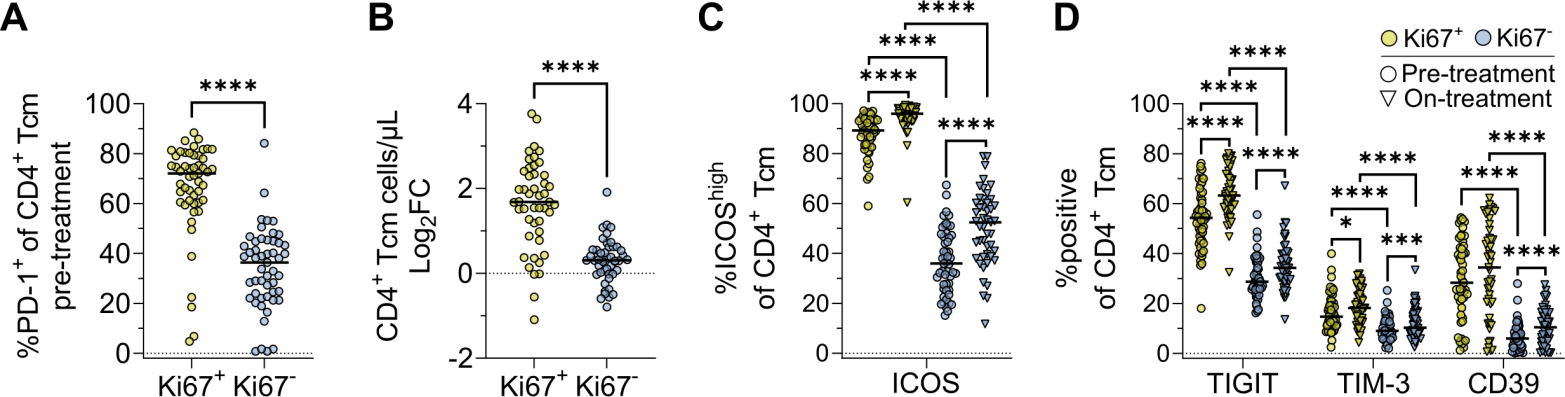
Combined immune checkpoint blockade enhances an already functional pool of Ki67^+^ cells. (**A**) PD-1 co-expression by Ki67^+^ and Ki67^−^ CD4^+^ Tcm cells. (**B**) Pre-treatment to on-treatment log_2_ fold change in absolute number of Ki67^+^ and Ki67^−^ CD4^+^ Tcm cells. (**C**) Ki67^+^ and Ki67^−^ CD4^+^ Tcm ICOS and (**D**) inhibitory marker expression. Plots show medians. Data shown from 51 pre-treatment and 47 on-treatment samples. Unpaired and paired statistical comparisons were performed with the Mann-Whitney and Wilcoxon signed-rank tests, respectively; Bonferroni correction for multiple comparisons was applied to all tests. *p<0.0167, **p<0.01, ***p<0.001, ****p<0.0001. CD39, cluster of differentiation 39; ICOS, inducible co-stimulator of T cells; log_2_FC, log_2_ fold change; PD-1, programmed cell death protein-1; Tcm, central memory T cell; TIGIT, T cell immunoreceptor with Ig and ITIM domains; TIM-3, T-cell immunoglobulin and mucin-domain containing-3.

Given the association between expansions of Ki67^+^ CD4^+^ Tcm cells and improved treatment outcome, we queried what phenotype these cells had prior to treatment in comparison to their Ki67^−^ counterparts, and if cICB induced additional functional maturation. Since most phenotypic changes were shared between responders and non-responders, we compared Ki67^+^ and Ki67^−^ CD4^+^ Tcm phenotypes for all patients. Significantly more Ki67^+^ T cells expressed PD-1 than their Ki67^-^ counterparts (median 72.1% vs 36.4%, respectively, p<0.0001) (figure 4A), and Ki67^+^ Tcm expanded 195% on-treatment, from a median of 6 to 17 cells/µL, compared with 51% for Ki67^−^ Tcm, from 171 to 257 cells/µL (figure 4B). Hence, through high pre-treatment expression of PD-1, Ki67^+^ cells are poised to respond to cICB. Congruent with this, significantly higher proportions of Ki67^+^ CD4^+^ Tcm cells expressed the activation marker ICOS both pre-treatment (89.3%) and on-treatment (96.0%) than Ki67^−^ CD4^+^ Tcm (pre=36.0%, p<0.0001; on=52.4%, p<0.0001) (figure 4C). Interestingly, pre-treatment Ki67^+^ CD4^+^ Tcm were also enriched for TIGIT (54.4% vs 28.7% in Ki67^−^ cells, p<0.0001), TIM-3 (14.7% vs 9.0%, p<0.0001), and CD39 (28.3% vs 5.9%, p<0.0001), and these frequencies increased only marginally on-treatment (figure 4D). While cytokine expression data could not be evaluated directly on Ki67^+^ and Ki67^−^ cells, total CD4^+^ Tcm cells predominantly expressed TNF-α and IL-2, with lower amounts of IFN-γ and negligible granzyme B. CD4^+^ Tcm from non-responders tended to have decreased expression of IFN-γ, TNF-α, and IL-2 on-treatment, while responders maintained equivalent expression to pre-treatment (online supplemental figure 7H). Hence, Ki67^+^ CD4^+^ Tcm appears to be an infrequent but highly functional subset present before cICB commencement that expands with only minor further activation and inhibitory marker expression in patients who respond to treatment.

### Severe toxicity is associated with higher proportions of TIM-3^+^ CD8^+^ T cells on treatment

**Figure 5:**
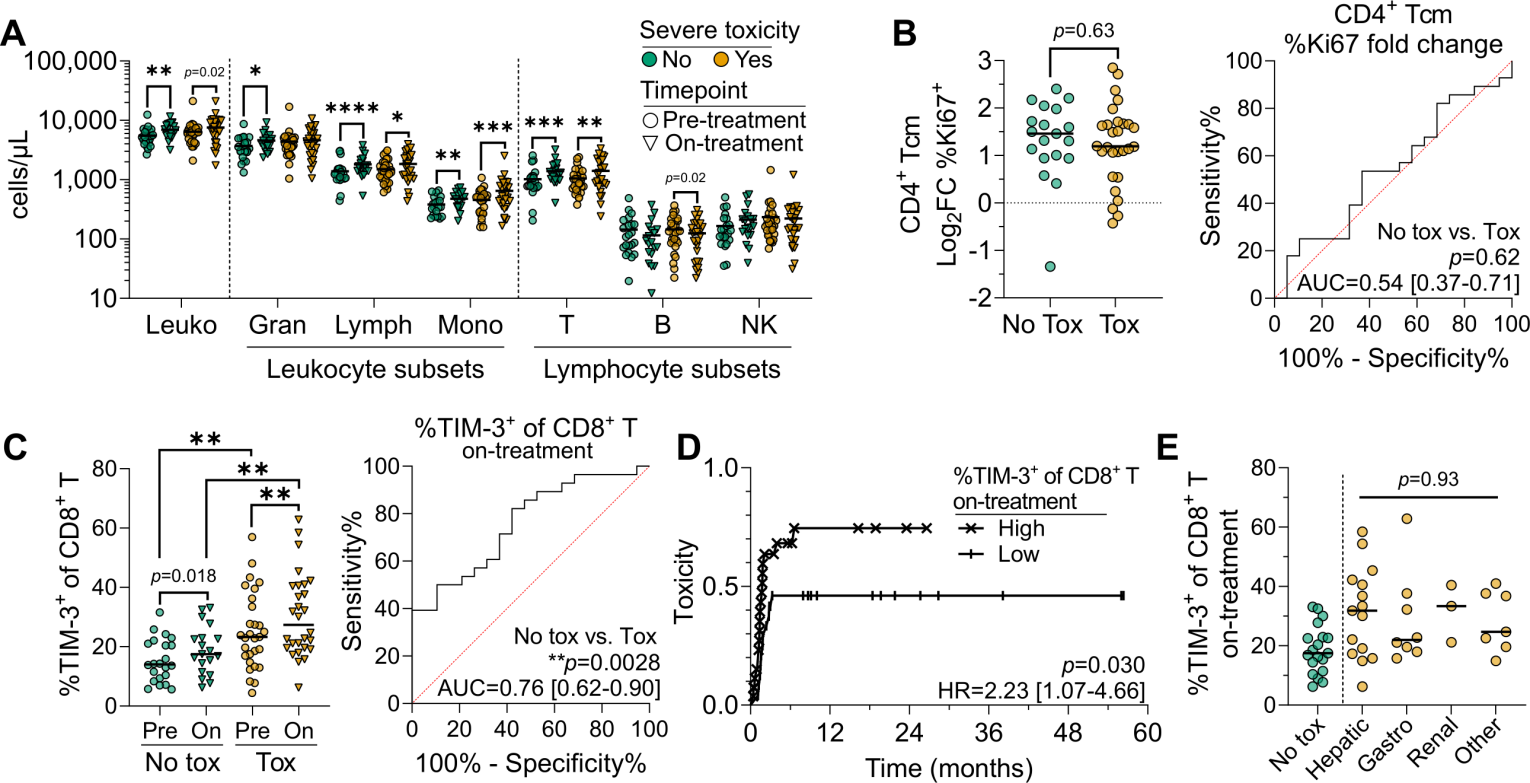
Higher TIM-3 expression is associated with the occurrence of severe toxicity. (**A**) Pre-treatment and on-treatment absolute counts of major innate and adaptive immune lineages in patients with or without severe (grade 3+) treatment-related toxicity. (**B**) Pre-treatment to on-treatment log_2_ fold change in Ki67 positivity within CD4^+^ Tcm cells, and associated ROC curve for patients with or without severe toxicity. Area under the curve (AUC) and 95% CI listed on plot. (**C**) Pre-treatment and on-treatment frequency of TIM-3^+^ cells within total CD8^+^ T cells, and associated on-treatment ROC curve for patients with or without severe toxicity. (**D**) Kaplan-Meier toxicity-free survival in patients with high (>median) or low (<median) frequency of on-treatment TIM-3^+^ CD8^+^ T cells. (**E**) TIM-3 frequency within CD8^+^ T cells in patients without severe toxicity, or with severe hepatic, gastrointestinal, renal, or other toxicity. Plots show medians. Data from patients with severe toxicity includes 29 pre-treatment and 28 on-treatment samples; data from patients without toxicity includes 22 pre-treatment and 19 on-treatment samples. Statistical comparisons were performed with the Mann-Whitney test; Bonferroni correction for multiple comparisons was applied to all tests. *p<0.0167, **p<0.01, ***p<0.001, ****p<0.0001. log_2_FC, log_2_ fold change; NK, natural killer; ROC, receiver operator characteristic; Tcm, central memory T cell; TIM-3, T-cell immunoglobulin and mucin-domain containing-3.

In our cohort, 29 patients (57%) experienced severe toxicity, with a median time to onset of 46 days post-commencement of ICB, ranging from 12 to 198 days, with hepatic (16/29 patients) and gastrointestinal (8/29 patients) complications being most common. Five patients experienced severe toxicity affecting two organ sites. No associations were observed between severe toxicity and previous treatment (p>0.99), ECOG status (p=0.25), sex (p=0.55), or age (p=0.89). Pre-treatment counts or on-treatment dynamics of major immune lineages and subsets did not differ significantly between patients with or without severe toxicity (figure 5A). Similarly, Treg abundance was equivalent both pre-treatment and on-treatment (online supplemental figure 8A). In contrast to response outcomes, CD4^+^ Tcm Ki67 dynamics did not associate with the onset of severe toxicity (AUC=0.54, 95% CI 0.37 to 0.71, p=0.62) (figure 5B, online supplemental figure 8B). Rather, the immune feature that associated most significantly with severe toxicity was the frequency and abundance of TIM-3^+^ CD8^+^ T cells pre-treatment and on-treatment (figure 5C, online supplemental figure 8C). TIM-3 frequency within CD8^+^ T cells increased from 19.7% to 22.5% in patients without severe toxicity on-treatment, and from 23.6% to 30.1% in patients with severe toxicity. On-treatment, TIM-3 frequency significantly differentiated toxicity groups (AUC=0.76, 95% CI 0.62 to 0.90, p=0.0028) (figure 5C), and remained significant after adjusting for pre-treatment ECOG performance score (p=0.0072) (online supplemental figure 8E). Further, patients with a TIM-3^+^ CD8^+^ T-cell frequency above the median cohort value of 22.5% had significantly shorter time to toxicity onset (figure 5D). Similar dynamics were observed for the absolute abundance of TIM-3^+^ CD8^+^ T cells (online supplemental figure 8C,D). While TIM-3 expression was most abundant on CD8^+^ Tnaive at both pre-treatment and on-treatment time points (46.8% and 44.0%, respectively), it increased in CD8^+^ Tscm, Tcm, TemRO, and TemRA cells on-treatment (online supplemental figure 8F), and was only significantly different between toxicity groups on CD8^+^ TemRO (p=0.011) and TemRA (p=0.025) populations on-treatment. CD8^+^ TemRO cells in patients with toxicity also more frequently co-expressed ICOS (p=0.016) and Ki67 (p=0.015) (online supplemental figure 8G). While statistical power was limited when comparing organ-specific toxicity subgroups, CD8^+^ T-cell TIM-3 expression did not vary significantly by toxicity site (figure 5E). Hence, distinct immunological changes appear associated with the onset of toxicity and the response to treatment.

## Discussion

We sought to address the unmet need for predictive biomarkers of cICB response and toxicity through profiling of pre-treatment and early on-treatment systemic immunity from a cohort of patients with advanced melanoma. Through detailed evaluation of the T-cell compartment we identified that higher on-treatment Ki67 upregulation in CD4^+^ Tcm was associated with better response to treatment and longer PFS, while patients experiencing severe toxicity had a higher frequency of TIM-3 expression within CD8^+^ T cells.

We additionally found that one cycle of cICB recovered systemic T-cell counts through expansion of memory and regulatory subsets, and this correlated significantly with pre-treatment PD-1 expression. This aligns with the notion that cICB efficacy relies on existing immune responses,^39 51^ and that PD-1^+^ CD8^+^ T cells are the major responding population to cICB.^19^ While previous studies have found that increased memory T-cell abundance associates with improved response to ICB,^21 37 38^ the data here suggest that memory T-cell expansion is a pharmacodynamic marker of treatment effect and is agnostic of response. These expanded populations co-expressed high levels of both activation and inhibitory markers, but we found that cICB enriched for proliferative effector cells over Treg only in responders, supporting work suggesting that the balance of effector and regulatory subsets is important for treatment outcome.^23^ Further, the dynamic balance of activation and inhibitory markers on critical cell subsets, such as cytotoxic and exhausted CD8^+^ T cells, is likely an important determinant of cICB outcome. Recent reports have shown that cICB expands TCF1^−^ CD127^−^ Tex, partially through promotion of TCF1^+^ CD127^+^ Tpex differentiation.^18 19^ While our panel did not contain TCF1 to definitively identify Tpex/Tex subsets, expanded CD8^+^ TemRO/RA cells upregulated markers of activation (ICOS, Ki67), inhibition (CTLA-4, TIGIT, TIM-3, and CD39), and the transcription factors Tbet and TOX, indirectly supporting the notion that cICB promotes the differentiation of Tpex towards transitory/intermediate and terminal Tex states.^26 52^ Importantly, these transitory/intermediate Tex cells maintained some effector functionality, and in support of this, we found no downregulation of key cytokines including granzyme B, IFN-γ, or TNF-α. Interestingly, we observed that non-responder CD8^+^ TemRO cells tended to have elevated pre-treatment TOX expression compared with healthy controls and responders, implying that non-responder Tex cells may be more terminally differentiated prior to commencing cICB and thus less amenable to reinvigoration. TOX expression in CD8^+^ TemRO cells of responders increased to equivalent levels on-treatment, a shift that was also mirrored in the proportion of total CD127^−^ CD8^+^ T cells. Similar to reports in patients with metastatic renal cell carcinoma treated with cICB, we observed a relative enrichment of eosinophils over neutrophils in responders on-treatment,^12^ but did not see an association between NER and OS.^11^ LMR and NLR ratios, as well as absolute lymphocyte counts, also did not associate with response or OS in this cohort.

While innate and CD8^+^ T cell dynamics broadly failed to differentiate responders and non-responders robustly, the magnitude of Ki67 upregulation in CD4^+^ Tcm cells significantly discriminated response groups and tended to associate with longer PFS. This emphasizes the importance of evaluations beyond the CD8^+^ T-cell compartment, and for assessing the role of CD4^+^ T cells in cICB efficacy, which may both help CD8^+^ T responses and exert direct antitumor activity.^53 54^ While we hypothesize that this feature is indicative of overall immune activation rather than the sole effector cell of cICB, Ki67^+^ CD4^+^ Tcm cells had an activated/exhausted phenotype pre-treatment, with elevated ICOS, PD-1, TIGIT, TIM-3, and CD39, and these markers increased further on-treatment and to a greater extent than their Ki67^−^ counterparts. These cells may thus be a cICB CD4^+^ equivalent to the PD-1^+^ CD8^+^ T cells that provide the “proliferative burst” during PD-1 blockade and are important for treatment efficacy.^27 28^ Indeed, Ki67^+^ T cells, particularly CD4^+^ T cells, were enriched in responders to cICB across multiple tumor types,^36^ and a small cohort study recently identified that patients with advanced melanoma responding to cICB had a larger on-treatment expansion of peripheral CD4^+^ T cells, particularly Tcm, over non-responders.^38^ Findings from patients with head and neck squamous cell cancer suggest that cICB induces CD4^+^ Tcm cells to traffic from tumor-draining lymph nodes via the blood to the tumor, where they differentiate to effector memory and Th1 cells.^55^ Notably, the majority of immunogenic tumor neoantigens are recognized by CD4^+^, not CD8^+^, T cells,^56^ and CD4^+^ Th1 effector cells have been shown to indirectly kill major histocompatibility complex-I deficient and IFN-unresponsive tumors.^57^ We here observed that responders had an absolute expansion of IFN-γ^+^ Th1 cells, suggesting a functional mechanism through which expanded CD4^+^ Tcm cells may contribute to improved antitumor responses. Further, the development of cytolytic CD8^+^ T cells depends on CD4 T-cell help via IL-21,^25^ and the therapeutic efficacy of CTLA-4 blockade is reliant on IL-21 signaling, which is increased during cICB.^19^ These data highlight the functional importance of CD4^+^ T cells for antitumor responses during cICB, and position Ki67 upregulation in CD4^+^ Tcm as a functional readout of the antitumor immune response during cICB.

Previous studies have suggested numerous immune features that associate with toxicity, including increased activated CD4^+^ effector memory cell frequencies,^58^ serum IL-17A,^59^ and on-treatment Ki67^+^ cells within CD8^+^ Tcm,^60^ and decreased frequencies of Treg^33 61^ and various immune lineage counts and ratios.^14^ Importantly, Ki67 upregulation in CD4^+^ Tcm did not associate with severe toxicity; rather, severe toxicity was associated with an increased frequency and abundance of TIM-3^+^ CD8^+^ T cells. TIM-3 expression is lowered on T cells in patients with ulcerative colitis, multiple sclerosis, rheumatoid arthritis, and psoriasis, and TIM-3 blockade worsens inflammatory bowel disease and exacerbates autoimmunity in mouse models.^62^ The association between heightened TIM-3 expression on T cells and severe toxicity observed here suggests that these may be self-reactive cells normally constrained by inhibitory checkpoint signaling, which following cICB are no longer sufficiently controlled. Increased TIM-3 expression by CD8^+^ T cells in patients with toxicity appeared to be due to TemRO/RA cells, which also had moderately increased ICOS and Ki67 expression, and may support reports of increased frequencies of CD8^+^ effector memory cells in patients with toxicity during PD-1 monotherapy or cICB.^63 64^

We found that the most discriminatory features for both response and toxicity were observed on-treatment, suggesting that the changes induced by cICB are more important than pre-treatment immune state, a paradigm that has been observed previously.^4 21^ This reinforces the utility of blood-based biomarkers, which are more accessible than tissue samples once treatment has commenced and hence allow monitoring of on-treatment immune dynamics.

This work contains several limitations; notably, we had a relatively limited cohort size, but this was strengthened by paired pre-treatment and on-treatment data in a patient cohort with uniform treatment and cancer type. On-treatment samples were unavailable from four non-responders, skewing this time point towards responders. Additionally, we did not include anti-IgG4 to assess PD-1-bound nivolumab in on-treatment samples and hence could not assess on-treatment PD-1 expression, and our panel design precluded assessment of Ki67 co-expression with CTLA-4 and cytokine markers.

Nonetheless, this discovery project identified promising early on-treatment candidate biomarkers of response and toxicity for further prospective validation, and highlights the importance of assessing both CD4^+^ and CD8^+^ T-cell responses to cICB. We further propose that toxicity and response to cICB may be driven by distinct immune features, introducing the possibility of uncoupling these clinical outcomes and enabling safer treatments.

## Supplementary material

10.1136/jitc-2025-012317 online supplemental file 1

## Data Availability

Data are available upon reasonable request.
