# Supplementary material for: Expansion of a circulating Ki67-positive effector T-cell population following combined PD-1 and CTLA-4 blockade for melanoma is predictive of treatment response
Source: J Immunother Cancer. 2025 Oct 9;13(10):e012317. doi: 10.1136/jitc-2025-012317 (PMC12516982; doi:10.1136/jitc-2025-012317)
Supplement: online supplemental file 1 [file jitc-13-10-s001.pdf]

Supplementary table 1: TruCount antibody panel.

					Test use	
**Target**	**Fluorochrome**	**Clone**	**Vendor**	**RRID^**	**µL/test**	**µg mL^{-1}**
CD3	FITC	UCHT1	BD Biosciences	AB_395739	3	0.56
CD4	BV605	RPA-T4	BD Biosciences	AB_2744420	0.5	0.36
CD8	BV510	SKI	BD Biosciences	AB_2722546	0.5	1.43
CD14	APC-H7	MφP9	BD Biosciences	AB_1645725	1	0.36
CD16	PE	3GA	Biolegend	AB_314208	0.7	1.00
CD19	PE-Cy7	SJ25C1	BD Biosciences	AB_396893	1	0.71
CD45	PerCP-Cy5.5	2D1	BD Biosciences	AB_400194	4	0.34
CD56	PE	B159	BD Biosciences	AB_395906	5	1.79
HLA-DR	APC	L243	Biolegend	AB_314688	2	0.71

^RRID: Research resource identifier

Supplementary table 2: TruCount panel population definitions.

	**Population name**	**Phenotype definition**
1	Granulocytes	SSC^{high} CD45^{+}
2	∟neutrophils	SSC^{high} CD45^{+} CD16/56^{+}
3	∟eosinophils	SSC^{high} CD45^{+} CD16/56^{-}
	Mononuclear cells	SSC^{inter} CD45^{+}
5	∟M-DC	SSC^{inter} CD45^{+} CD3^{-} CD19^{-} HLA-DR^{+} CD16/56^{-} CD14^{-}
6	∟Monocytes	SSC^{inter} CD45^{+} CD3^{-} CD19^{-} HLA-DR^{+} CD16/56$^{+/-}$ CD14$^{+/-}$
7	∟classical	SSC^{inter} CD45^{+} CD3^{-} CD19^{-} HLA-DR^{+} CD16/56^{-} CD14^{+}
8	∟intermediate	SSC^{inter} CD45^{+} CD3^{-} CD19^{-} HLA-DR^{+} CD16/56^{+} CD14^{+}
9	∟non-classical	SSC^{inter} CD45^{+} CD3^{-} CD19^{-} HLA-DR^{+} CD16/56^{+} CD14^{-}
10	Lymphocytes	SSC^{low} CD45^{+}
11	∟B cells	SSC^{low} CD45^{+} CD3^{-} CD19^{+}
12	∟NK cells	SSC^{low} CD45^{+} CD3^{-} CD16/56^{+}
13	∟T cells	SSC^{low} CD45^{+} CD19^{-} CD3^{+}
14	∟CD4^{+} T cells	SSC^{low} CD45^{+} CD19^{-} CD3^{+} CD4^{+} CD8^{-}
15	∟CD8^{+} T cells	SSC^{low} CD45^{+} CD19^{-} CD3^{+} CD4^{-} CD8^{+}

Supplementary table 3: Spectral panel 1 (resting) and panel 2 (activated) reagents.

Target	Fluorochrome	Clone	Supplier	RRID^	Panel usage	Test use µL/test	µg mL^{-1}
CD3	BUV805	UCHT1	BD Biosciences	AB_2800945	1,2	1.25	2.5
CD4	cFluor YG584	SK3	Cytek Biosciences	AB_2870183	1,2	1.25	0.5
CD8	Spark Blue 550	SK1	Biolegend	AB_2885083	1,2	0.25	0.1
CD16	BV510	3GA	BD Biosciences	AB_2819982	1	1.25	5
CD19	BUV563	SJ25C1	BD Biosciences	AB_2744296	1,2	0.3	0.6
CD25	PE-Fire700	M-A251	Biolegend	AB_2870202	1,2	2.5	2.5
CD39	APC-Fire750	A1	Biolegend	AB_2876678	1	0.5	0.5
CD45	PerCP	HI30	Biolegend	AB_2650838	1,2	0.5	2
CD45RA	BUV496	HI100	BD Biosciences	AB_893341	1,2	0.04	0.0064
CD56	BUV737	B159	BD Biosciences	AB_2874456	1	0.4	1.6
CD57	PB	HNK-1	Biolegend	AB_2871176	1	0.2	0.2
CD95	PE-Dazzle594	DX2	Biolegend	AB_2562458	1,2	0.625	1.25
CD127	R718	HIL-7R-M21	BD Biosciences	AB_2564221	1,2	2.5	2.5
CCR7	BV750	GO43H7	Biolegend	AB_2869977	1,2	2.5	10
CTLA-4	BV785	BNI3	Biolegend	AB_2810582	2	0.6	1.2
CXCR5	BV605	RF8B2	BD Biosciences	AB_2740110	1	2.5	10
EOMES	PE-Cy7	WD1928	ThermoFisher	AB_2573456	1	2.5	0.6
GrzmB	BV510	GB11	BD Biosciences	AB_2738174	2	0.3	1.2
ICOS	BUV661	DX29	BD Biosciences	AB_2871056	1	1.25	5
IFNγ	BV605	B27	BD Biosciences	AB_2737926	2	0.6	1.2
IL-2	AF488	MQ1-17H12	BD Biosciences	AB_2738566	2	2.5	2.5
IL-4	APC	MP4-25D2	Biolegend	AB_2295923	2	2.5	0.2
IL-10	BV421	JES3-9D7	BD Biosciences	AB_493368	2	2.5	2.5
IL-17A	PE-Cy7	BL168	Biolegend	AB_315131	2	0.2	0.6
IRF4	AF647	IRF4.3E4	Biolegend	AB_2564047	1	1.25	2.5
Ki67	BUV395	B59	BD Biosciences	AB_2738577	1	1.25	7.5
KLRG1	APC	2F1	Biolegend	AB_10645509	1	0.3	0.3
PD-1	BV786	EH12.1	BD Biosciences	AB_2738425	1	1.25	5
Tbet	PE-Cy5	4B10	ThermoFisher	AB_2815071	1	0.4	1.6
TCRγδ	PerCP-Vio700	REA591	Miltenyi Biotec	AB_2733074	1,2	1	8
TIGIT	BV421	A5153G	Biolegend	AB_2632925	1	2.5	2.5
TIM-3	BB515	7D3	BD Biosciences	AB_2744368	1	2.5	5
TNFα	BUV395	MAb11	BD Biosciences	AB_2738533	2	0.5	2
TOX	PE	TXRX10	ThermoFisher	AB_10855034	1	0.625	2.5
Viability	Live/Dead Blue	L34962	ThermoFisher	*NA*	1,2	1.25	*NA*

^Research resource identifier

Supplementary table 4: Panel 1 (resting) population definitions.

	Population name	Phenotype definition
	All populations gated from live lymphocytes: $CD45^{+}$ SSC^{low} Live/Dead $Blue^{-}$	
1	B cells	$CD3^{-}$ $CD19^{+}$
2	NK cells	$CD3^{-}$ $CD19^{-}$ $CD16/CD56^{+}$
3	T cells	$CD3^{+}$ $CD19^{-}$
4	∟ $TCR\gamma\delta^{+}$	$CD3^{+}$ $CD19^{-}$ $TCR\gamma\delta^{+}$
7	∟ $TCR\alpha\beta^{+}$	$CD3^{+}$ $CD19^{-}$ $TCR\gamma\delta^{-}$
10	∟ $CD4^{-}$ $CD8^{-}$	$CD3^{+}$ $CD19^{-}$ $TCR\gamma\delta^{-}$ $CD4^{-}$ $CD8^{-}$
11	∟ CD4 T cells	$CD3^{+}$ $CD19^{-}$ $TCR\gamma\delta^{-}$ $CD4^{+}$ $CD8^{-}$
12	∟ Treg	$CD3^{+}$ $CD19^{-}$ $TCR\gamma\delta^{-}$ $CD4^{+}$ $CD25^{+}$ $CD127^{-/lo}$
13	∟ NOT Treg	$CD3^{+}$ $CD19^{-}$ $TCR\gamma\delta^{-}$ $CD4^{+}$ $CD25^{+/lo}$ $CD127^{+/-}$
14	∟ Naive/stem-like	$CD3^{+}$ $CD19^{-}$ $TCR\gamma\delta^{-}$ $CD4^{+}$ $CD25^{+/lo}$ $CD127^{+/-}$ $CD45RA^{+}$ $CCR7^{+}$
15	∟ Tnaive	$CD3^{+}$ $CD19^{-}$ $TCR\gamma\delta^{-}$ $CD4^{+}$ $CD25^{+/lo}$ $CD127^{+/-}$ $CD45RA^{+}$ $CCR7^{+}$ $CD95^{-}$
16	∟ Tscm	$CD3^{+}$ $CD19^{-}$ $TCR\gamma\delta^{-}$ $CD4^{+}$ $CD25^{+/lo}$ $CD127^{+/-}$ $CD45RA^{+}$ $CCR7^{+}$ $CD95^{+}$
17	∟ Tcm	$CD3^{+}$ $CD19^{-}$ $TCR\gamma\delta^{-}$ $CD4^{+}$ $CD25^{+/lo}$ $CD127^{+/-}$ $CD45RA^{-}$ $CCR7^{+}$
18	∟ TemRO	$CD3^{+}$ $CD19^{-}$ $TCR\gamma\delta^{-}$ $CD4^{+}$ $CD25^{+/lo}$ $CD127^{+/-}$ $CD45RA^{-}$ $CCR7^{-}$
19	∟ TemRA	$CD3^{+}$ $CD19^{-}$ $TCR\gamma\delta^{-}$ $CD4^{+}$ $CD25^{+/lo}$ $CD127^{+/-}$ $CD45RA^{+}$ $CCR7^{-}$
20	∟ Tfh	$CD3^{+}$ $CD19^{-}$ $TCR\gamma\delta^{-}$ $CD4^{+}$ $CD25^{+/lo}$ $CD127^{+/-}$ $CD45RA^{+}$ $CXCR5^{+}$
21	∟ CD8 T cells	$CD3^{+}$ $CD19^{-}$ $TCR\gamma\delta^{-}$ $CD8^{+}$ $CD4^{-}$
22	∟ Naive/stem-like	$CD3^{+}$ $CD19^{-}$ $TCR\gamma\delta^{-}$ $CD8^{+}$ $CD45RA^{+}$ $CCR7^{+}$
23	∟ Tnaive	$CD3^{+}$ $CD19^{-}$ $TCR\gamma\delta^{-}$ $CD8^{+}$ $CD25^{+/lo}$ $CD127^{+/-}$ $CD45RA^{+}$ $CCR7^{+}$ $CD95^{-}$
24	∟ Tscm	$CD3^{+}$ $CD19^{-}$ $TCR\gamma\delta^{-}$ $CD8^{+}$ $CD25^{+/lo}$ $CD127^{+/-}$ $CD45RA^{+}$ $CCR7^{+}$ $CD95^{+}$
25	∟ Tcm	$CD3^{+}$ $CD19^{-}$ $TCR\gamma\delta^{-}$ $CD8^{+}$ $CD45RA^{-}$ $CCR7^{+}$
26	∟ TemRO	$CD3^{+}$ $CD19^{-}$ $TCR\gamma\delta^{-}$ $CD8^{+}$ $CD45RA^{-}$ $CCR7^{-}$
27	∟ TemRA	$CD3^{+}$ $CD19^{-}$ $TCR\gamma\delta^{-}$ $CD8^{+}$ $CD45RA^{+}$ $CCR7^{-}$

Supplementary table 5: Panel 2 (activated) population definitions.

	**Population name**	**Phenotype definition**
	All populations gated from live lymphocytes:	CD45^{+} SSC^{low} Live/Dead Blue^{-}
1	T + NK cells	CD19^{-}
2	∟ TCRγδ^{+}	CD19^{-} TCRγδ^{+}
3	∟ TCRγδ^{-}	CD19^{-} TCRγδ^{-}
4	∟ CD4^{+} T cells	CD19^{-} TCRγδ^{-} CD4^{+} CD8^{-}
5	∟ Treg	CD19^{-} TCRγδ^{-} CD4^{+} CD25^{+} CD127$^{-/lo}$
6	∟ NOT Treg	CD19^{-} TCRγδ^{-} CD4^{+} CD25$^{+/lo}$ CD127$^{+/-}$
7	∟ Naive/stem-like	CD19^{-} TCRγδ^{-} CD4^{+} CD25$^{+/lo}$ CD127$^{+/-}$ CD45RA^{+} CCR7^{+}
8	∟ Tnaive	CD19^{-} TCRγδ^{-} CD4^{+} CD25$^{+/lo}$ CD127$^{+/-}$ CD45RA^{+} CCR7^{+} CD95^{-}
9	∟ Tscm	CD19^{-} TCRγδ^{-} CD4^{+} CD25$^{+/lo}$ CD127$^{+/-}$ CD45RA^{+} CCR7^{+} CD95^{+}
10	∟ Tcm	CD19^{-} TCRγδ^{-} CD4^{+} CD25$^{+/lo}$ CD127$^{+/-}$ CD45RA^{-} CCR7^{+}
11	∟ TemRO	CD19^{-} TCRγδ^{-} CD4^{+} CD25$^{+/lo}$ CD127$^{+/-}$ CD45RA^{-} CCR7^{-}
12	∟ TemRA	CD19^{-} TCRγδ^{-} CD4^{+} CD25$^{+/lo}$ CD127$^{+/-}$ CD45RA^{+} CCR7^{-}
13	∟ Th1	CD19^{-} TCRγδ^{-} CD4^{+} CD25$^{+/lo}$ CD127$^{+/-}$ IL-17A^{-} IFNγ^{+}
14	∟ Th2	CD19^{-} TCRγδ^{-} CD4^{+} CD25$^{+/lo}$ CD127$^{+/-}$ IL-17A^{-} IFNγ^{-} IL-4^{+}
15	∟ Th17	CD19^{-} TCRγδ^{-} CD4^{+} CD25$^{+/lo}$ CD127$^{+/-}$ IL-17A^{+} IFNγ^{-}
16	∟ CD8^{+} T cells	CD19^{-} TCRγδ^{-} CD8^{+} CD4^{-}
17	∟ Naive/stem-like	CD19^{-} TCRγδ^{-} CD8^{+} CD25$^{+/lo}$ CD127$^{+/-}$ CD45RA^{+} CCR7^{+}
18	∟ Tnaive	CD19^{-} TCRγδ^{-} CD8^{+} CD25$^{+/lo}$ CD127$^{+/-}$ CD45RA^{+} CCR7^{+} CD95^{-}
19	∟ Tscm	CD19^{-} TCRγδ^{-} CD8^{+} CD25$^{+/lo}$ CD127$^{+/-}$ CD45RA^{+} CCR7^{+} CD95^{+}
20	∟ Tcm	CD19^{-} TCRγδ^{-} CD8^{+} CD45RA^{-} CCR7^{-}
21	∟ TemRO	CD19^{-} TCRγδ^{-} CD8^{+} CD45RA^{-} CCR7^{-}
22	∟ TemRA	CD19^{-} TCRγδ^{-} CD8^{+} CD45RA^{+} CCR7^{-}

Supplementary table 6: Pre- to on-treatment Log_2 fold change in absolute T cell subset abundance.

	Responders		Non-responders		
**Population**	**Log_2FC**	***p*-value (from pre-treatment)**	**Log_2FC**	***p*-value (from pre-treatment)**	***p*-value (R vs. NR)**
$CD8^+$ Total	0.32	0.00059	0.19	0.034	0.7
$CD8^+$ Tnaive	-0.14	0.063	-0.03	0.82	0.27
$CD8^+$ Tscm	0.13	0.29	0.02	0.56	0.71
$CD8^+$ Tcm	0.11	0.055	0.13	0.27	0.89
$CD8^+$ TemRO	0.88	5.3×10^{-6}	0.39	0.00043	0.17
$CD8^+$ TemRA	0.30	0.0048	0.19	0.083	0.7
$CD4^+$ Total	0.33	0.00064	0.47	0.0052	0.57
$CD4^+$ Tnaive	-0.16	0.012	0.08	0.71	0.082
$CD4^+$ Tscm	-0.05	0.57	0.14	0.67	0.46
$CD4^+$ Tcm	0.37	0.00013	0.40	0.0027	0.63
$CD4^+$ TemRO	1.12	3.1×10^{-8}	0.86	6.1×10^{-5}	0.68
$CD4^+$ TemRA	0.42	0.0031	0.40	0.083	0.85
$CD4^+$ Treg	0.65	8.4×10^{-7}	0.73	0.00076	0.63

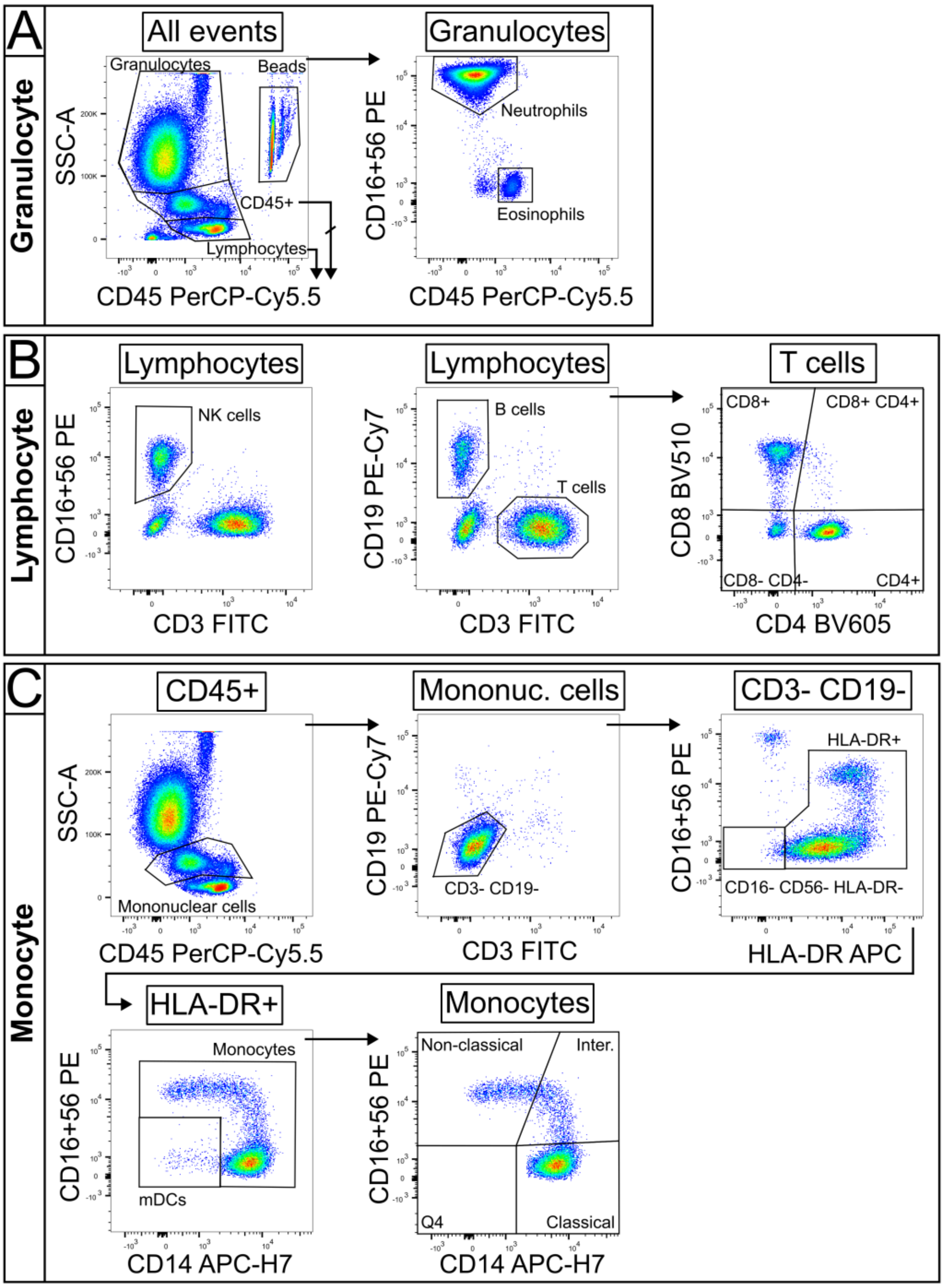

Supplementary figure 1: Gating scheme for TruCount analysis of whole blood.

Whole blood was stained in a Lyse/No Wash protocol and analyzed on either an LSR II or FACSLyric (BD Biosciences). For FACSLyric acquisition (pictured), a CD45 threshold was set to exclude debris. (A) Major leukocyte populations were gated based on CD45 expression; granulocytes (CD45$^+$ SSC^{hi}), lymphocytes (CD45$^+$ SSC^{lo}) and TruCount beads (CD45^{hi}), and granulocytes were separated into neutrophils (CD16$^+$) and eosinophils (CD16$^-$). (B) Lymphocytes were separated into NK cells (CD3$^-$ CD16/56$^+$), B cells (CD3$^-$ CD19$^+$) and T cells (CD3$^+$ CD19$^-$). T cells were further characterized based on CD4 and CD8 expression. (C) Mononuclear cells were sequentially gated to remove contaminating cells and separated into myeloid dendritic cells (mDC; CD16/56$^-$ CD14$^-$ HLA-DR$^+$) and monocytes (HLA-DR$^+$). Monocytes were classified as classical (CD14$^+$ CD16/56$^-$), intermediate (CD14$^+$ CD16/56$^+$) and non-classical (CD14$^-$ CD16/56$^+$). HLA-DR expression heatmap overlay was used to assist monocyte subset gating (not shown).

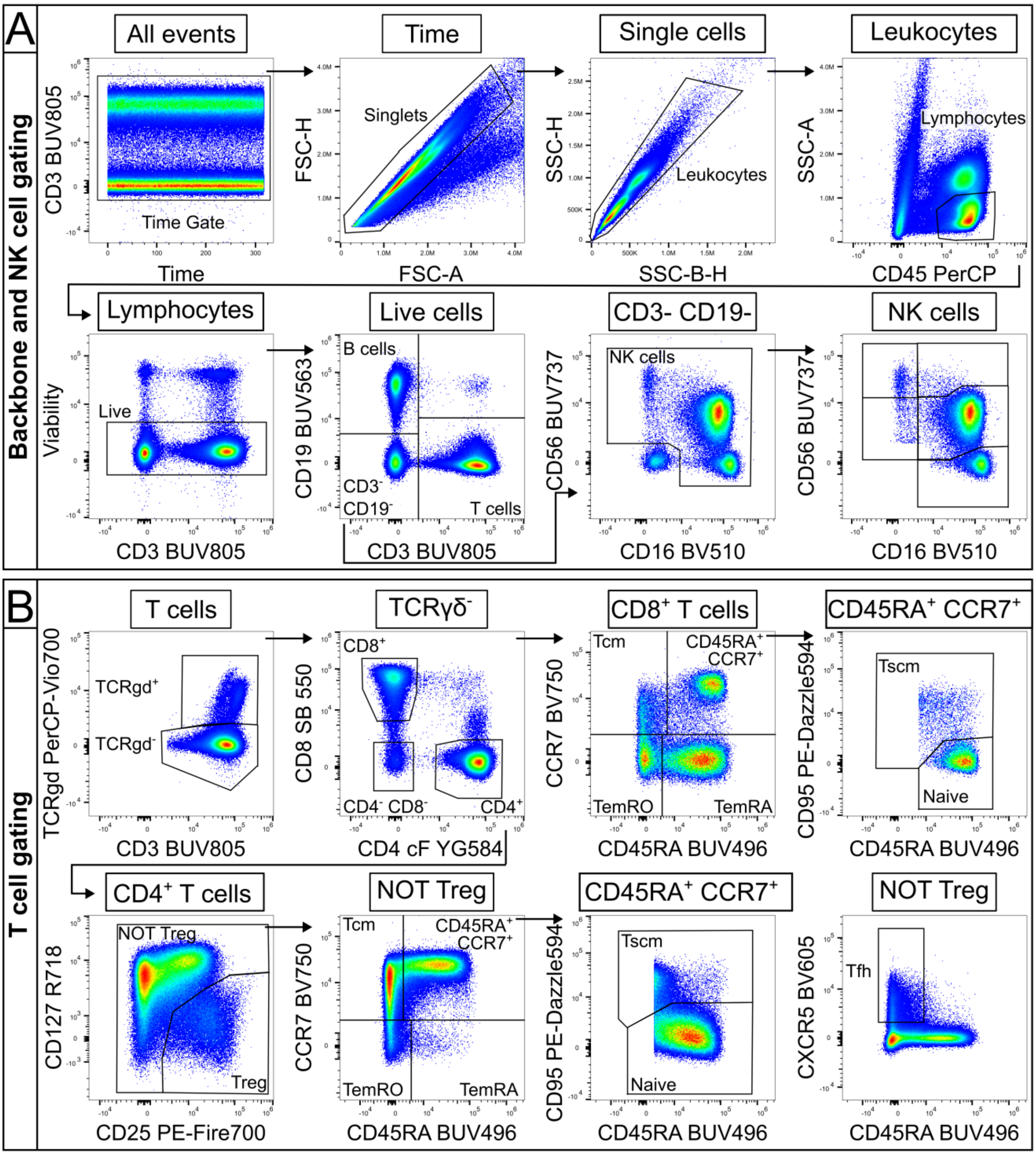

Supplementary figure 2. Gating scheme for the 27-color resting panel to interrogate T and NK cells for surface and intracellular markers.

(A) Single, live lymphocytes were gated sequentially before division into B cells (CD19+), T cells (CD3+) and NK cells (CD19- CD3- CD16/56+). NK-cell subsets were further separated based on differential expression of CD16 and CD56. (B) TCRγδ+ T cells were defined before CD4 and CD8 lineage gating on the TCRγδ- subset. CD4+ Treg cells (CD25+ CD127-/lo) were excluded before gating of memory populations from the NOT Treg gate. Memory populations for both CD4+ and CD8+ T cells were defined as naive (CD45RA+ CCR7+ CD95lo), stem cell-like memory (Tscm; CD45RA+ CCR7+ CD95hi), central memory (Tcm; CD45RA- CCR7+), Effector Memory CD45RO (TemRO; CD45RA- CCR7-) and effector memory CD45RA (TemRA; CD45RA+ CCR7-). Circulating CD4+ Tfh cells were defined as CD45RA- CXCR5+.

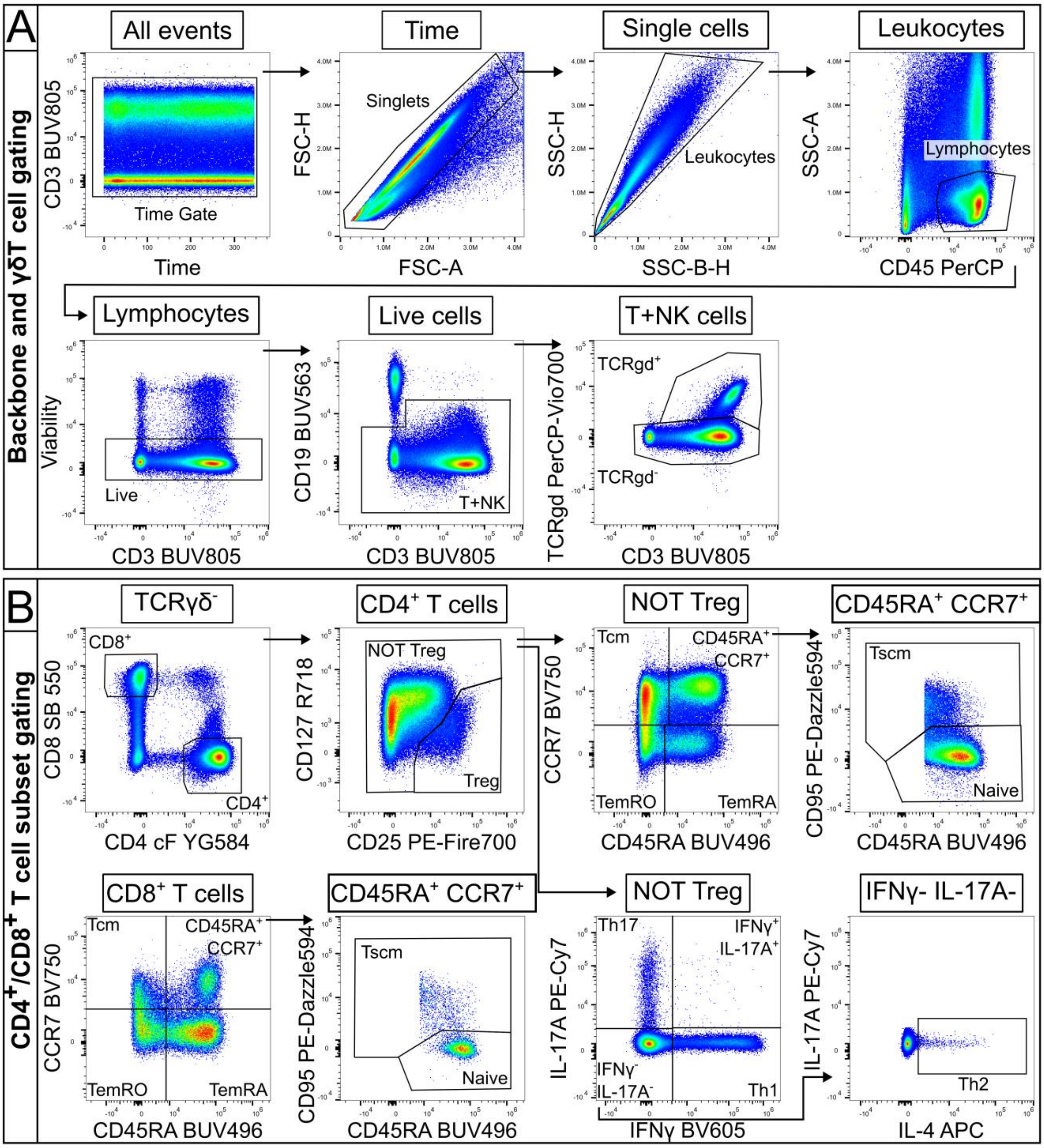

Supplementary figure 3. Gating scheme for the 20-color activated panel designed to interrogate cytokine production from CD3+CD28-stimulated T cells.

(A) Events were gated sequentially to select single, live lymphocytes. Due to CD3 down-regulation in some samples, T cells were gated from a combined T and NK cell gate (CD19$^-$ CD3$^{+/-}$) before gating of TCRγδ$^+$ cells. (B) CD4$^+$ and CD8$^+$ T cell subsets were gated as CD19$^-$ TCRγδ$^-$ CD4$^+$ or CD19$^-$ TCRγδ$^-$ CD8^{hi}, respectively (NK cells are TCRγδ$^-$ CD8$^{-/lo}$ CD4$^-$). Treg were gated as CD25$^+$ CD127$^{-/lo}$, with subsequent CD4$^+$ T memory and Th-cell gating from the 'NOT Treg' gate. Th cell subsets were defined by canonical cytokine expression (Th1, IFNγ$^+$; Th17, IL-17A$^+$; and Th2, IFNγ$^-$ IL-17A$^-$ IL-4$^+$). CD4$^+$ and CD8$^+$ T-cell memory subsets were defined by differential expression of CD45RA, CCR7 and CD95 as in the resting panel.

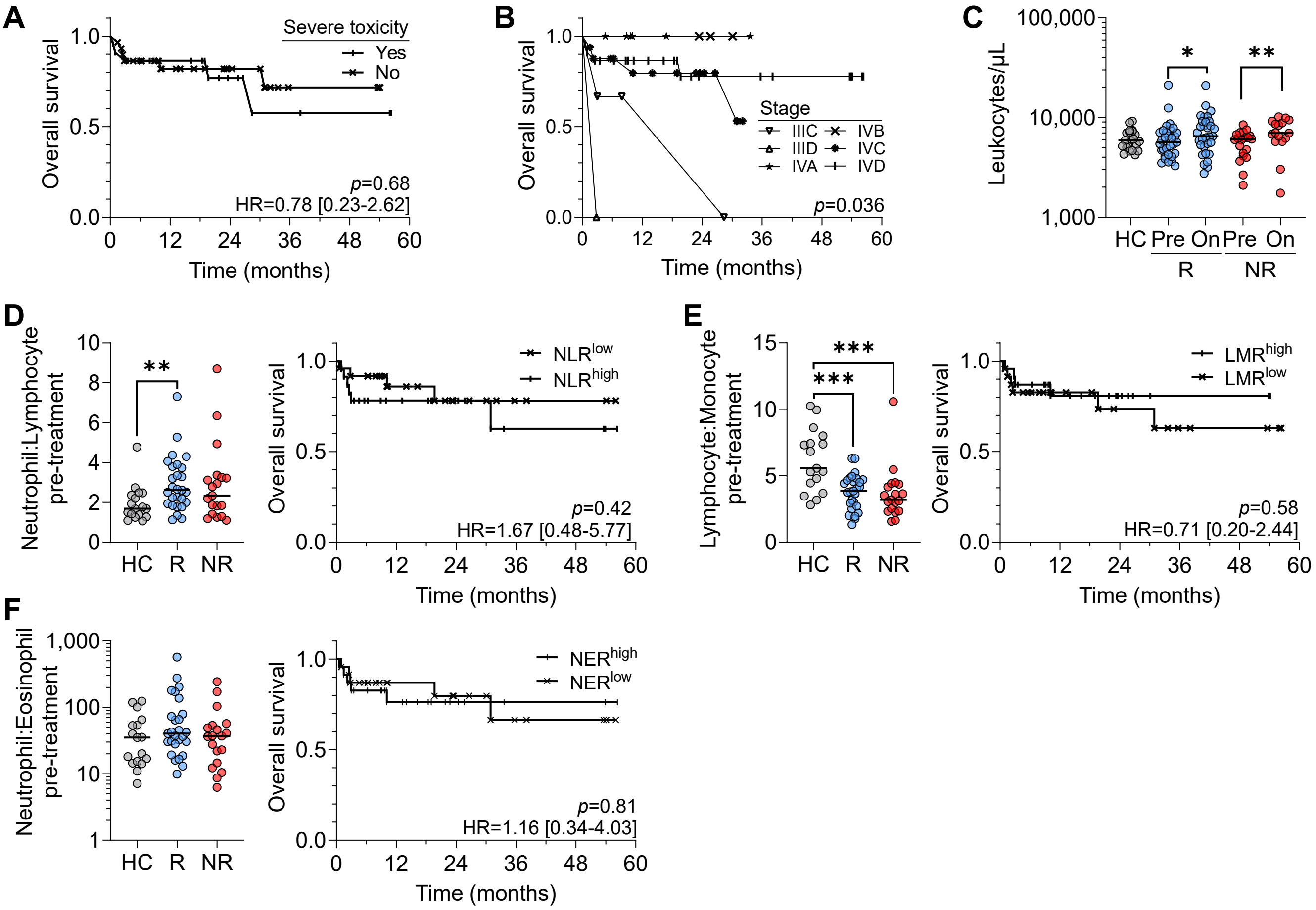

Supplementary figure 4: Pre-treatment characteristics and association with overall survival.

Kaplan-Meier survival curve and (A) occurrence of severe (grade 3+) toxicity and (B) stage at commencement of treatment. (C) Pre- and on-treatment leukocyte count in healthy controls (HC), responders (R), and non-responders (NR).. (D-F) Pre-treatment neutrophil to lymphocyte (NLR), lymphocyte to monocyte (LMR), and neutrophil to eosinophil (NER) ratios, and associated Kaplan-Meier survival curves for patients dichotomised by high (>median) or low (<median) ratio values. Plots show medians. Unpaired statistical comparisons were performed with the Mann-Whitney test and paired comparisons with the Wilcoxon signed-rank test. *$p<0.0167$, **$p<0.01$, ***$p<0.001$, ****$p<0.0001$.

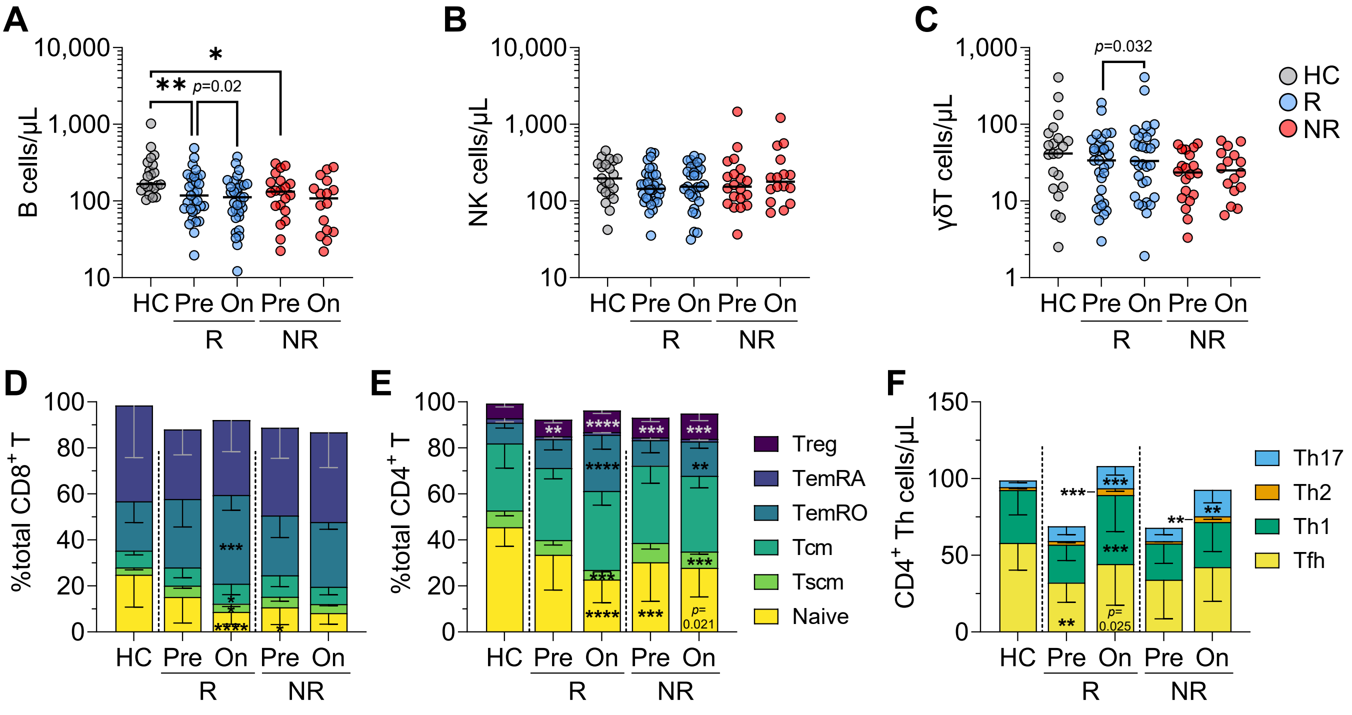

Supplementary figure 5: On-treatment changes to lymphocyte populations.

Pre- and on-treatment count of (A) B cells, (B) NK cells, and (C) γδT cells. (D) Proportional subset abundance within total $CD8^+$ and (E) $CD4^+$ T cells. (F) T helper (Th) 1 (Th1), Th2, Th17, and T follicular helper (Tfh) abundance. Plots show medians ± IQR. Unpaired statistical comparisons were performed with the Mann-Whitney test and paired comparisons with the Wilcoxon signed-rank test. *p<0.0167, **p<0.01, ***p<0.001, ****p<0.0001.

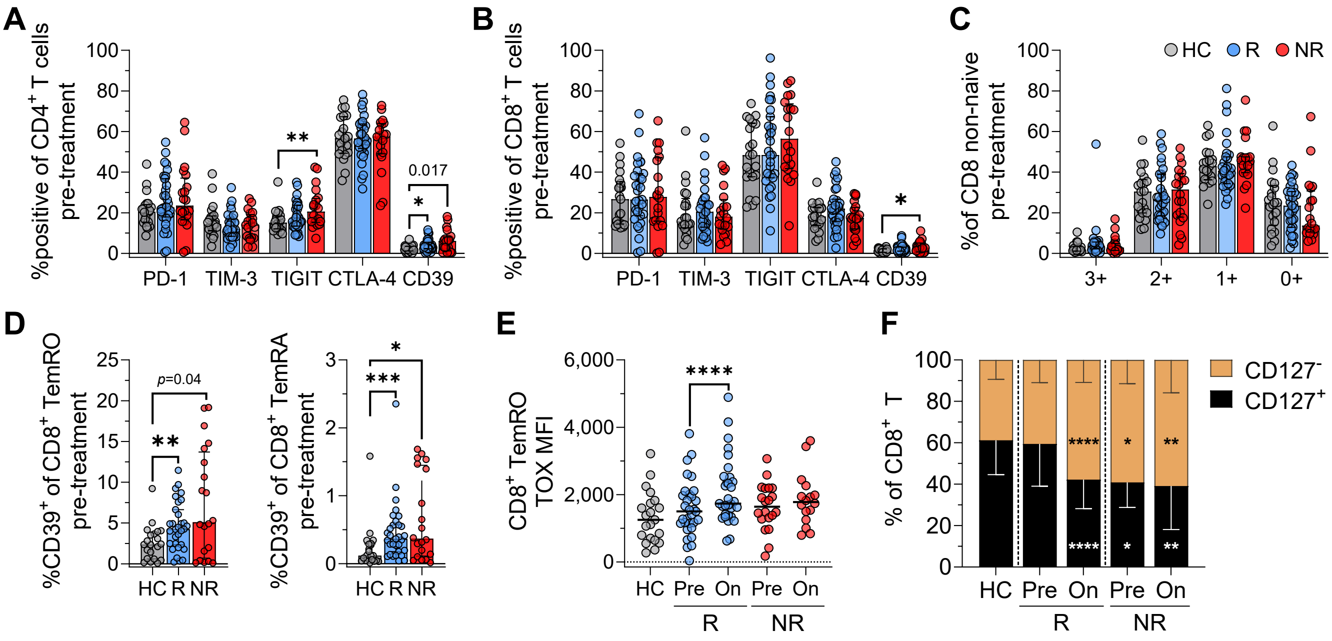

Supplementary figure 6: Inhibitory receptor expression and CD8^{+} T cell phenotype.

(A) Pre-treatment expression of inhibitory receptors on total CD4^{+} and (B) CD8^{+} T cells. (C) Pre-treatment Boolean co-expression of PD-1, TIM-3, and TIGIT on non-naive CD8^{+} T cells. (D) Pre-treatment expression of CD39 by CD8^{+} TemRO and TemRA cells. (E) Pre - and on-treatment TOX expression (median fluorescence intensity, MFI) by CD8^{+} TemRO cells. (F) CD127 expression by total CD8^{+} T cells. Plots show medians ± IQR. Unpaired statistical comparisons were performed with the Mann-Whitney test and paired comparisons with the Wilcoxon signed-rank test. *$p<0.0167$, **$p<0.01$, ***$p<0.001$, ****$p<0.0001$.

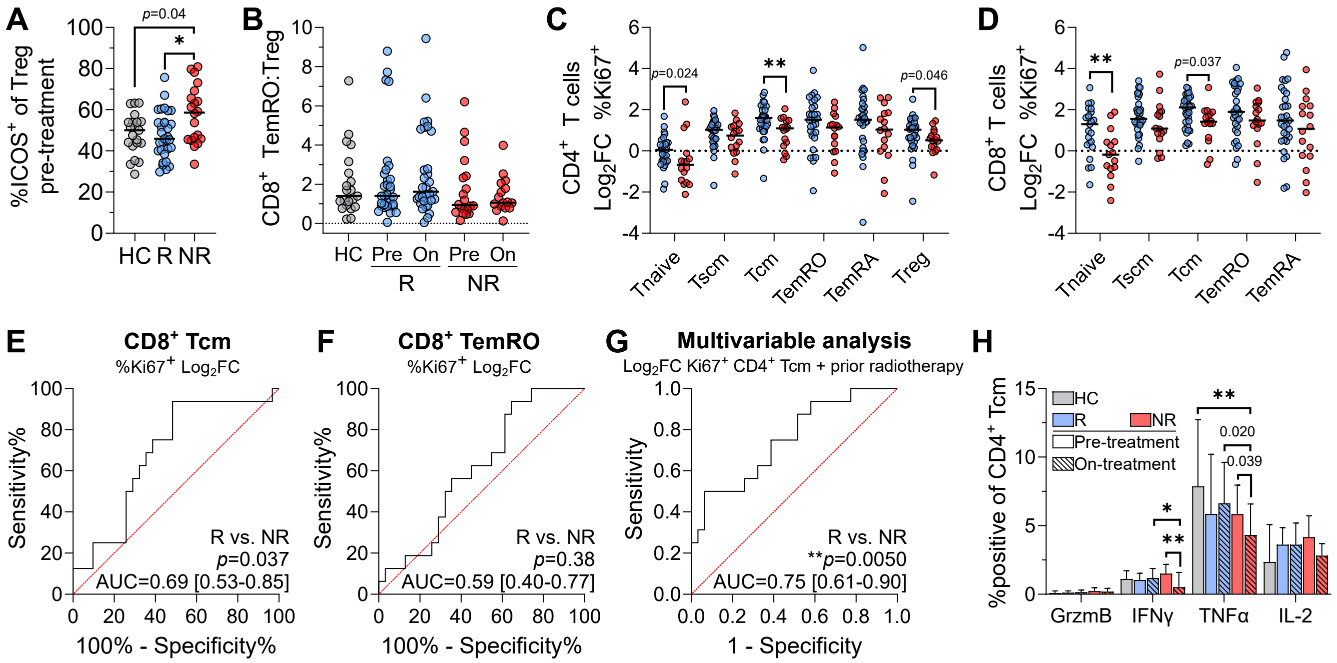

Supplementary figure 7: Treg, Tcm, and Ki67^{+} T-cell phenotype.

(A) Pre-treatment ICOS expression by Treg in healthy controls (HC), responders (R), and non-responders (NR). (B) Pre- and on-treatment ratio of CD8^{+} TemRO to Treg cell counts. Pre- to on-treatment Log_2 fold change in %Ki67 expression by (C) CD4^{+} and (D) CD8^{+} T cells. Responder vs. non-responder Receiver Operator Characteristic (ROC) curves with area under the curve (AUC) and 95% confidence intervals for (E) Log_2FC in %Ki67^{+} within CD8^{+} Tcm, (F) Log_2FC in %Ki67^{+} within CD8^{+} TemRO, and (G) multiple logistic regression survival plot incorporating Log_2FC in %Ki67^{+} within CD4^{+} Tcm and prior radiotherapy. (H) Pre- and on-treatment expression of granzyme B (GrzmB), interferon-gamma (IFNγ), tumour necrosis factor alpha (TNFα), and interleukin-2 (IL-2) by healthy controls (HC), responders (R), and non-responders (NR). Plots show medians. Unpaired statistical comparisons were performed with the Mann-Whitney test and paired comparisons with the Wilcoxon signed-rank test. *p<0.0167, **p<0.01, ***p<0.001, ****p<0.0001.

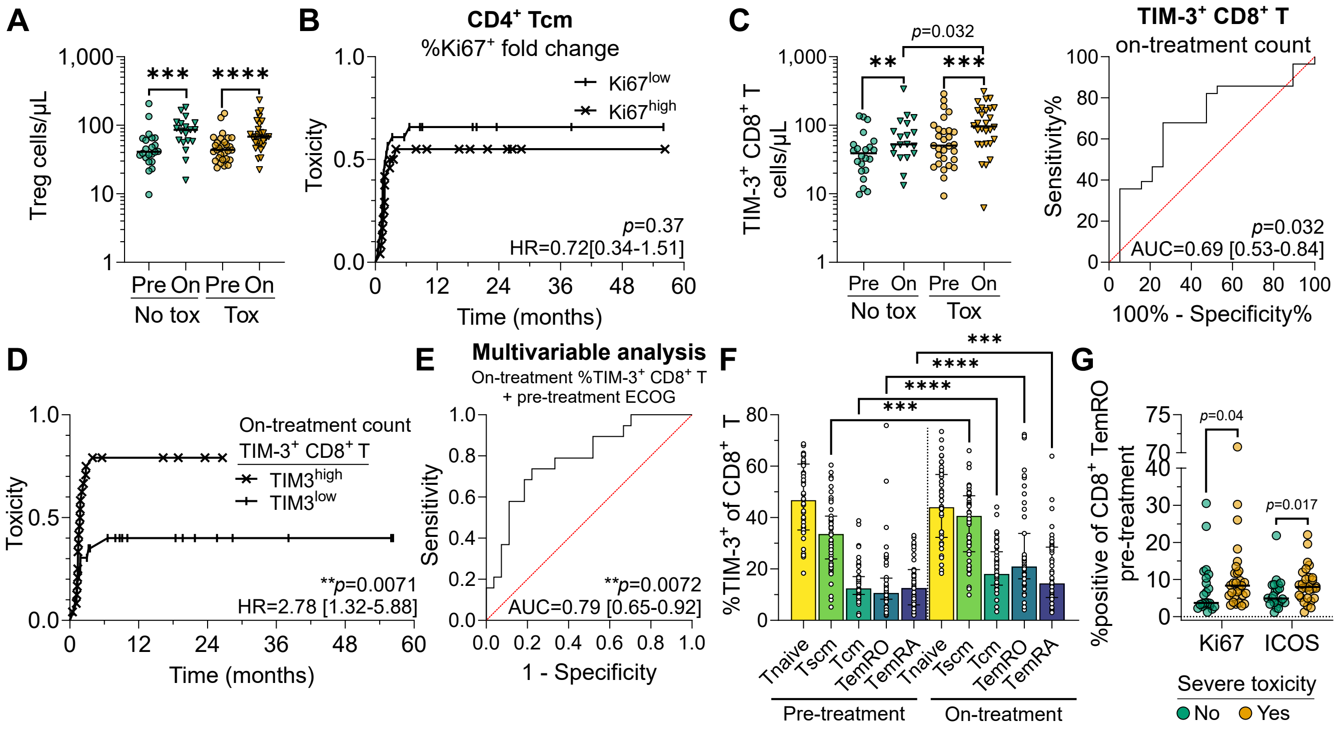

Supplementary figure 8: Immune cell correlates of severe toxicity.

(A) Pre- and on-treatment Treg abundance in patients with or without severe toxicity. (B) Kaplan-Meier toxicity for patients with high (>median) or low (<median) CD4$^+$ Tcm %Ki67$^+$ Log$_2$FC. (C) Absolute count of pre- and on-treatment TIM-3$^+$ CD8$^+$ T cells, and on-treatment ROC curve for patients with or without severe toxicity. Area under the curve (AUC) and 95% confidence interval listed. (D) Kaplan-Meier toxicity-free survival in patients with high (>median) or low (<median) on-treatment TIM-3+ CD8+ T cell count. (E) Multiple logistic regression survival plot incorporating CD8$^+$ T cell %TIM-3$^+$ frequency and pre-treatment ECOG performance score. (F) TIM-3 expression in CD8$^+$ T cell subsets pre- and on-treatment in all patients. (G) Ki67 and ICOS expression in CD8$^+$ TemRO cells pre-treatment. Plots show medians. Paired pre- to on-treatment comparisons used the Wilcoxon signed-rank test. *p<0.0167, **p<0.01, ***p<0.001, ****p<0.0001.
